# Process evaluation of school-based high-intensity interval training interventions for children and adolescents: a systematic review and meta-analysis of randomized controlled trials

**DOI:** 10.1186/s12889-024-17786-6

**Published:** 2024-02-02

**Authors:** Yong Liu, Curtis A. Wadey, Alan R. Barker, Craig A. Williams

**Affiliations:** Children’s Health and Exercise Research Centre, Public Health and Sports Sciences, Faculty of Health and Life Sciences, University of Exeter, Heavitree Road, Exeter, Devon EX1 2LU UK

**Keywords:** Process evaluation, High-intensity interval training, School, Children and adolescents

## Abstract

**Background:**

Several systematic reviews have been published to investigate the effectiveness of high-intensity interval training (HIIT) in schools. However, there has been limited attention given to understanding the functioning of the intervention processes, which is of paramount importance for interpreting and translating the intervention effectiveness. The aim of this systematic review is to determine the extent to which process evaluation is measured in school-based HIIT interventions and to explore the effects of process evaluation and intervention characteristics on cardiorespiratory fitness (CRF), body composition, muscular strength, and blood pressure.

**Methods:**

A comprehensive search was conducted in SPORT Discus (EBSCOhost), Web of Science, Scopus, Medline (Ovid) and Cochrane Central Register of Controlled Trials. The extent to which process evaluation is measured was narratively reported, alongside with the guidance of process evaluation of complex interventions by UK Medical Research Council. Meta-analyses and meta-regressions were conducted to determine the effects of process evaluation and intervention characteristics to the intervention outcomes.

**Results:**

The literature search identified 77 studies reporting on 45 school-based HIIT interventions. In total, five interventions reported process evaluation in a section or in a separate study, and only one intervention adopted a process evaluation framework. On average, 6 out of 12 process evaluation measures were reported in all interventions. Subgroup analyses did not indicate any beneficial treatment effects for studies with process evaluation group, whereas all pooled data and studies without process evaluation group showed significant improvement for CRF and body composition.

**Conclusion:**

Process evaluation is frequently omitted in the literature of school-based HIIT in children and adolescents. Although reporting of process evaluation measures may not directly associate with better intervention outcomes, it allows accurate interpretation of intervention outcomes, thereby enhancing the generalisability and dissemination of the interventions.

**Supplementary Information:**

The online version contains supplementary material available at 10.1186/s12889-024-17786-6.

## Introduction

Physical activity (PA) is well documented in promoting physical fitness and health, including improvement in body composition, cardiorespiratory fitness (CRF), musculoskeletal function and psychological health in children and adolescents [1–4]. Despite the importance of PA, it is reported that less than 20% of adolescents meet the World Health Organization guideline of an average of 60 min moderate to vigorous PA (MVPA) per day [3]. In addition, recent studies have shown that the COVID-19 pandemic, local or international conflicts, and economic and climate changes, have further exacerbated global physical inactivity [5, 6].

Schools, where children and adolescents spend most of their waking time, play a fundamental role in promoting MVPA amongst children and adolescents [7]. Time in PA participation can be accumulated not only during physical education classes, but also throughout various breaks or even in academic classes [8]. However, the effectiveness of school-based interventions to increase engagement in MVPA is limited [1, 9, 10]. Commonly cited barriers for MVPA engagement are time constraints, lack of motivation and facilities [11, 12]. Consequently, adopting a time-efficient and engaging PA strategy may be a promising approach for health promotion within school settings.

Despite concerns for high-intensity interval training (HIIT) to be safely performed by inactive population, the elderly, or patients [13], emerging evidence suggests that HIIT is a form of exercise that is safe [14], time-efficient [11] and enjoyable [15] to be performed among healthy school-aged children and adolescents. Apart from these allures, HIIT provides young people with opportunities to engage in vigorous PA (VPA) [14], which is favourably associated with several cardiometabolic health markers (e.g., CRF) in youth [16, 17]. Therefore, the popularity of tailoring HIIT in school-based health-promoting interventions has grown in recent years. Indeed, several school-based HIIT reviews have been published [18–23], supporting beneficial effects of HIIT on health markers such as body composition, CRF and neuromuscular performance in comparison to a control group (e.g., maintaining daily living, attending regular physical education classes etc.).

However, the question remains as to how practitioners and researchers should accurately interpret and capitalise on these promising findings and whether the effectiveness of these interventions are transferable to different contexts. These important questions will not necessarily be addressed if an intervention is conducted in isolation, without reporting the implementation process [24]. Process evalution provides insights into how an intervention is conducted, drawing upon the causality underpinning the treatment effects [25]. By incorporating process evaluation with randomised controlled trials (RCTs), gold standard for establishing intervention effectiveness [26], the implementation fidelity and quality of the RCTs can be assessed and the causal mechanisms and contextual factors shaping the intervention effectiveness can be clarified [24]. Therefore, process evaluation is complementary to that of RCTs by not only assessing if the intervention works, but also why it works and if it works in other contexts [24], thereby informing future intervention maintenance, scaling up and transfer [25].

Recognising the growing importance of process evaluation, a cascade of frameworks emerged under the term ‘process evaluation’ [24, 27–29]. Among them, the Medical Research Council (MRC) guidance on the process evaluation of complex interventions, developed by Moore et al., stands out as particularly comprehensive [24]. This framework drew upon insights related to the existing definition of process evaluation and was developed by a group of researchers with expertise in complex interventions through a series of workshops, conferences, and seminars. The MRC guidance delineates three domains of process evaluation: implementation, mechanisms of impact and context. These domains encompass understandings of what and how the intervention is implemented; how the intervention brings changes to the intervention outcomes; and how the context shapes the implementation process and outcomes. Notably, the MRC guidance has been adopted in reviews of different areas [30, 31]. For example, Ma and colleagues adapted the framework to assess the extent to which process evaluation is reported in interventions aimed at improving gross motor competence in children and adolescents [30].

While process evaluation serves as an important complement to outcome assessment, its reporting remains infrequent and insufficient [32]. Among the six school-based HIIT reviews [18–23], none has primarily focused on process evaluation. Despite one study reported certain aspects of process evaluation measures, such as fidelity and attendance [18], comprehensive attention to this critical aspect is notably lacking. With more studies starting to report process evaluation in school-based HIIT RCTs [33–37], a systematic review assessing process evaluation is timely in synthesising the evidence and providing recommendations for future interventions. Therefore, the primary aim of this systematic review is to examine the extent of process evaluation reporting in school-based HIIT studies. The secondary aim is to determine the effects of process evaluation and intervention characteristics on CRF, body composition, muscular health, and blood pressure.

## Methods

This review aligns with the guidelines of Preferred Reporting Items for Systematic Reviews and Meta-Analyses (PRISMA) [38], see Additional File 1 for the PRISMA checklist, and Cochrane Handbook for Systematic Reviews of Interventions [39]. The protocol of the review was registered on PROSPERO (CRD42022314567).

### Inclusion and exclusion criteria 

#### Population 

To be included in this review, participants needed to be 5–18-year-old school children or adolescents with no restrictions placed on weight status. However, studies which focused on specific sub-populations, such as youth athletes or paediatric disease/disability groups (e.g., diabetes mellitus) were excluded.

#### Intervention

Intervention duration ≥ 2 weeks was considered eligible, and the intervention must comprise at least one HIIT treatment group to be included in the review. HIIT in the current study was defined as repeating short (within 45 s) to long (up to 4 min) bouts of high-intensity exercises (e.g., 85% maximum heart rate (HR_max_)), interspersed with rest or recovery periods [40].

#### Comparator 

Any form of control or comparative groups were included for assessing the extent of process evaluation reporting. However, only the studies with a usual practice control group (e.g., continued with regular physical education session) were included in the subsequent meta-analyses and meta-regressions considering the heterogeneity among comparative exercise groups.

#### Outcome

The primary aim was to explore and report the extent of process evaluation in school-based HIIT interventions. Consequently, no restriction was made in terms of intervention outcomes. However, only the following outcomes were considered for meta-analysis and regression: CRF, body composition, muscular strength, and blood pressure. These variables were selected as they are the most frequently studied fitness parameters in the literature of school-based HIIT interventions [18, 22].

#### Study design

The interventions must be conducted on school premises, regardless of where the outcome data were collected. In addition, only RCTs were included in the current study since it is considered as the gold standard for establishing intervention effectiveness [26].

### Search strategy and selection

A comprehensive search for the relevant literature was conducted in SPORTDiscus (EBSCOhost), Web of Science, Scopus, Medline (Ovid) and Cochrane Central Register of Controlled Trials from inception to March 2022. Search strategy was formulated based upon the guideline of Peer Review of Electronic Search Strategy (PRESS) [41] and was checked with an information specialist before commencement. The full search strategy is available in Additional File 2.

Upon removal of duplicates on Endnote (Clarivate Analytics, Philadelphia, USA), two reviewers (YL and CW) independently screened the titles and abstracts against the inclusion and exclusion criteria in a blinded manner on Rayyan [42]. Subsequently, a discussion was organised to compare and reconcile the independent screening results, reaching a consensus on the papers to undergo a full-text review. This process was reiterated via another round of independent screening and discussion to complete the selection. Disagreements were resolved by discussion with two additional authors (CAW and ARB). The references cited in the included studies were manually checked for identifying additional eligible studies and an updated search was made in November 2022. Whenever not enough information in the manuscript for deciding, an inquiry email was sent to the authors for clarification. If the authors did not reply, these studies were listed as awaiting classification and were excluded if no responses were received after a second inquiry attempt at least fourteen days apart. These excluded papers are presented in Additional File 3.

### Data extraction

A data extraction sequential list (Table 1) was predefined in case multiple measurements of the same outcome emerged. The rationale for the prioritisation was based upon measurement properties (e.g., validity and reliability) and popularity of the measurements. Data extraction on process evaluation measures was guided by the Medical Research Council (MRC) process evaluation framework [24], including domains of implementation, mechanisms of impact, and context. The framework is elaborately conducted, providing a systematic, comprehensive, and exhaustive process evaluation review [25, 30]. A process evaluation framework by Ma et al. [30] was referred to adapt the framework into practice. The process evaluation measures were predefined in Table 2. Of note, although defined as session quality (e.g., attendance and dose received) and intensity in school-based HIIT studies [37, 43], fidelity was solely represented by intensity in the current review, as session quality was reported separately as other process evaluation measures (e.g., dose delivered).

**Table 1:** Preferential orders of data extraction for outcome variables

Sequence	CRF	Muscular strength	Body composition
1	VO_2max/peak_	Handgrip	% Body fat
2	Field-based tests	Standing long jump	Fat mass
3	Estimated VO_2peak_	Push-up	BMI/BMI-z
4	Others	Others	Others

*CRF* cardiorespiratory fitness, *VO*_*2max/peak*_ maximal/peak oxygen uptake, *BMI (-z)* body mass index (z score)

**Table 2:** Process evaluation concepts, applying and examples regarding process evaluation measures

Process evaluation measures	Concepts	Applying	Examples
**Implementation process**	The structures, resources and mechanisms through which delivery is achieved.	Studies intended to report process evaluation measures.	Study by Harris et al. [44] reported all the process evaluation measures.
**Implementation**: The process through which interventions are delivered, and what is delivered in practice.			
Fidelity	The consistency of what is implemented with the planned intervention.	How is the prescribed intensity achieved?	Participants wore Polar H7 heart rate monitors to monitor exercise intensity of 85% of heart rate maximum [33].
Reach	The extent to which a target audience comes into contact with the intervention.	How many schools or participants were contacted.	A total of 70 envelopes were delivered to potential participants from which 21 were returned [45].
Dose delivered	How much intervention is delivered.	HIIT session length, frequency and intervention duration/sessions in total.	53 mins per day and 4 times per week for 117 sessions in total [46].
Recruitment & retention	NA	How many participants randomised and how many of them completed the study.	154 participants were contacted, 29 agreed to participate, while 26 completed the intervention [47].
Adaptation	Alterations made to an intervention in order to achieve better contextual fit.	Changes been made to facilitate the high-intensity interval training interventions.	Employed rating of perceived exertion as a substitution of HR monitors [48].
**Mechanisms of impact**: The intermediate mechanisms through which intervention activities produce intended (or unintended) effects.			
Mediator	Intermediate processes which explain subsequent changes in outcomes.	Variables explored which mediate the intervention outcome variables.	Inactive adolescents showed significant improvements in well-being after intervention, while not in active adolescents [49].
Dose received	NA	How much intervention sessions were successfully delivered.	All the sessions were delivered as intended [35].
Unintended consequences	NG	Reporting of adverse event or other unanticipated fairs during the intervention.	The teachers were absent from the intervention owing to sickness [44].
Response	How participants interact with a complex intervention.	Feedbacks from the participants or deliverers' point of view, via interviews, questionnaires and so on.	Most of teachers and students reported they would like to continue the programme [50].
**Context**: Factors external to the intervention which may influence its implementation, or whether its mechanisms of impact act as intended.			
Barriers	NA	Contextual factors which undermine implementation, intervention mechanisms and outcomes	Time constrains was highlighted as a key barrier to long-term adherence [51].
Facilitators	NA	Contextual factors which facilitate implementation, intervention mechanisms and outcomes	A NZ$20 voucher was provided upon completion of the intervention [36].
Contamination	NA	Blinding made to avoid bias in allocating participants, performing HIIT or reporting data.	Testers were blind to group allocation. Control group was treated with placebo activities [52].

*NA* not available, *NG* not given

The following data were extracted: (1) key study characteristics (e.g., author name, publication year, participant characteristics, intervention details, and sample size); (2) process evaluation measures (implementation, mechanisms of impact and context); (3) post intervention (the closest to intervention endpoint) outcomes and results, including sample size, mean and standard deviation (SD) in intervention and non-exercise control groups. Where trials reported 95% confidence intervals [33, 50] or median and interquartile range [53], these were converted to means and SDs using established methods [54]. In addition, mean change values [50, 55] were extracted when post-means and SDs were not reported, and data were extracted from figures, via GetData Graph Digitizer, when not reported numerically [35]. Data extraction forms were developed, piloted, and refined through discussions across the authorship team. The extraction was conducted by YL and partially (30%) checked by CW for accuracy.

### Risk of bias assessment

The Cochrane risk-of-bias tool for randomized trials and revised Cochrane risk-of-bias tool for cluster-randomized trials [56] were adopted for quality assessment of RCTs and cluster-RCTs, respectively. Risk of bias for the outcomes of CRF, body composition and muscular strength were assessed separately from the following five domains: randomization process, intended interventions, missing outcome data, measurement of outcome and report of results by answering signalling questions. The judgments for each domain were summarised as “low risk of bias” “some concerns” or “high risk of bias”. An overall risk of bias judgement was reached through algorithms that map responses to signalling questions. The first author (YL) performed the risk of bias assessment, and the accuracy of the assessment was subsequently verified by a second author (CW) through a random sub-sample (30%) of studies, with less than 80% of consensus triggered a check for all the studies. Conflicts were discussed with consultation been made with either CAW or ARB whenever disputes occurred.

### Data synthesis and meta-analyses 

Process evaluation measures in relation to the included interventions were thematically assembled in line with the prescribed definition (Table 2) and were narratively reported. All the interventions were qualitatively synthesised. Random-effects meta-analyses were performed, incorporating subgroup analyses for studies with and without process evaluation, to determine the differences between the two groups concerning CRF, body composition, muscular strength and blood pressure. Where outcomes were reported using different measurement units, a standardised mean difference (SMD) effect size was reported. SMD was set as 0.2, 0.5 and 0.8, corresponding to small, medium and large effects [57]. Several sensitivity analyses were performed including a leave one out meta-analysis, the removal of high risk of bias studies, and studies that had a computed outcome score. A spectrum of intervention characteristics and process evaluation measures (Table 3) were selected and regressed to determine mediators of CRF, body composition, muscular strength and blood pressure. Heterogeneity was assessed via *I*^*2*^ and tau^2^ (*τ*^2^) statistic [39], publication bias was assessed via funnel plots and the Egger’s test [58]. The analyses were performed in STATA version 17 (College Station, Texas 77,845 USA) [59].

**Table 3:** Interpretation and coding regarding intervention characteristics and process evaluation measures

Mediator	Interpretation	Coding
**Intervention Characteristics**		
Randomisation	Studies randomised at individual or cluster level	0 = RCT, 1 = cluster RCT
Age	Average age of the participants. Median was taken if only an age range was available (e.g., 8–10 years = 9 years)	Continuous variable
Duration	Intervention duration in weeks. Where applicable, months were transferred to weeks by referring to the calendar	Continuous variable
Frequency	Sessions per week	0 = < 3, 1 = ≥ 3
Session length	Session length in minutes without warm up and cool down	Continuous variable
Work-to-rest ratio	Length of a work bout/length of a rest bout	0 = ratio < 1, 1 = ratio ≥ 1
Deliverer	Personnel who ran the intervention sessions	0 = researcher, 1 = teacher
Modality	Exercise modality performed during the sessions	0 = traditional (running or cycling), 1 = others
Occasion	Occasions when the interventions were carried out	0 = physical education classes, 1 = others
**Process Evaluation Measures**		
Overall	Number of process evaluation measures been reported	< 7 or ≥ 7 aspects been reported
Fidelity	How intensity been monitored	0 = not reported, 1 = reported
Recruitment and retention	Number of participants recruited and completed the intervention	0 = not reported, 1 = reported
Adaptation	Changes during intervention for facilitating the implementation	0 = not reported, 1 = reported
Mediator	Mediators been investigated which altered the outcome effects	0 = not reported, 1 = reported
Dose received	Doses been performed by participants	0 = not reported, 1 = reported
Response	Feedbacks from the deliverers and/or participants	0 = not reported, 1 = reported
Adverse event	Injury reported related to the intervention	0 = not reported, 1 = reported
Theoretical concept	Theoretical concept introduced to facilitate the intervention	0 = not reported, 1 = reported
Incentive	Strategies took to motivate participants	0 = not reported, 1 = reported
Training	Training courses prior to the intervention commencement	0 = not reported, 1 = reported
Barriers	Factors reported which undermined the intervention	0 = not reported, 1 = reported
Contamination	Blinding been taken to avoid impacts on outcome variables	0 = not reported, 1 = reported

*HIIT* high-intensity interval training, *RCT* randomised controlled trial

## Results

**Fig. 1:**
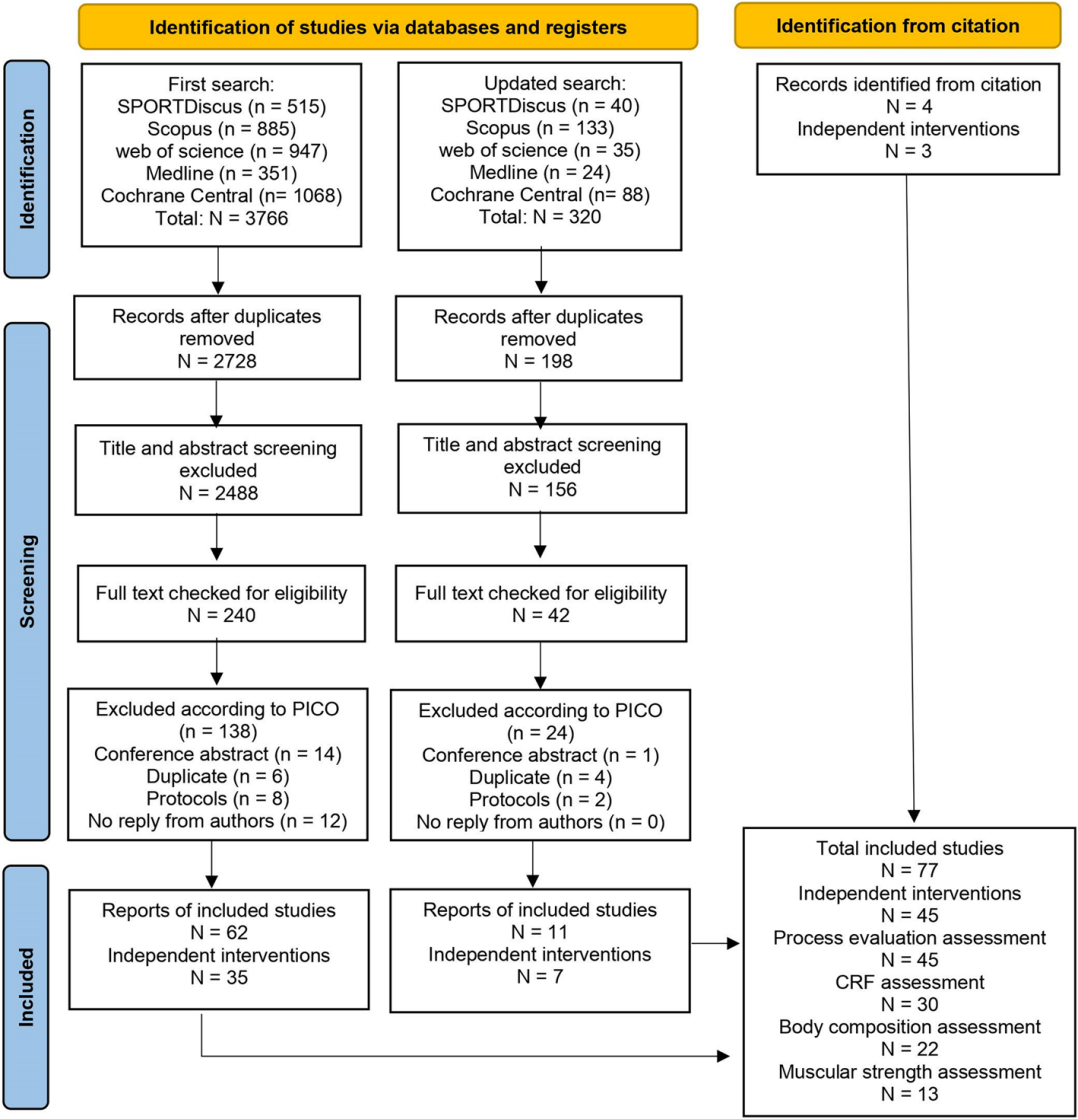
Preferred Reporting Items for Systematic Reviews and Meta-Analyses (PRISMA) flow diagram. PICO, population, intervention, comparison and outcomes; CRF, cardiorespiratory fitness

The initial search in March 2022 yielded 3,766 records, and an additional 320 records were identified in the updated search in November 2022. The PRISMA flowchart is shown in Fig. 1. The detailed reasons for the exclusion of the full text checked studies are presented in the Additional File 3. At length, 77 studies were included, covering 45 school-based HIIT interventions. All the interventions were included to determine the extent of process evaluation reporting. Of note, since only one study reported both blood pressure and process evaluation simultaneously [34], no further analysis was conducted for this outcome. Thus, 30, 22 and 13 interventions were included in meta-analyses and meta-regressions for CRF, body composition and muscular strength assessment, respectively.

### Study characteristics

In total, 15 interventions (33%) were cluster-RCTs. When combined, over 25,104 participants, across 17 Countries/regions, were included in this review. Twenty-one interventions (47%) were conducted in primary schools, while twenty-four interventions (53%) were delivered in secondary schools. The mean age was 12.2 years (*n* = 38), and 7 studies provided the age range of the participants only. There were 8 and 7 interventions targeted exclusively at female and male participants, respectively. Eleven interventions examined children living with overweight and obesity. The HIIT intervention duration ranged from 2 weeks to one academic year and was 12 weeks on average. The session frequency ranged from 1 to 5 times per week, with 3 (24 interventions, 53%) or 2 (14 interventions, 31%) sessions per week being most frequently reported. The HIIT sessions were as short as 4 min to as long as 43 min in total length. The work and rest intervals varied significantly, ranging from 10 s up to 240 s, with more than half of the interventions (*n* = 27) adopted a work interval less than 30 s. Additionally, the work-to-rest ratio was different from interventions, with the ratio = 1 (*n* = 17), < 1 (*n* = 5), > 1 (*n* = 9) or varied (*n* = 14). Twenty interventions (44%) adopted the traditional running or cycling modality. The details of all the intervention characteristics are summarised in the Additional File 4.

### Risk of bias

**Fig. 2:**
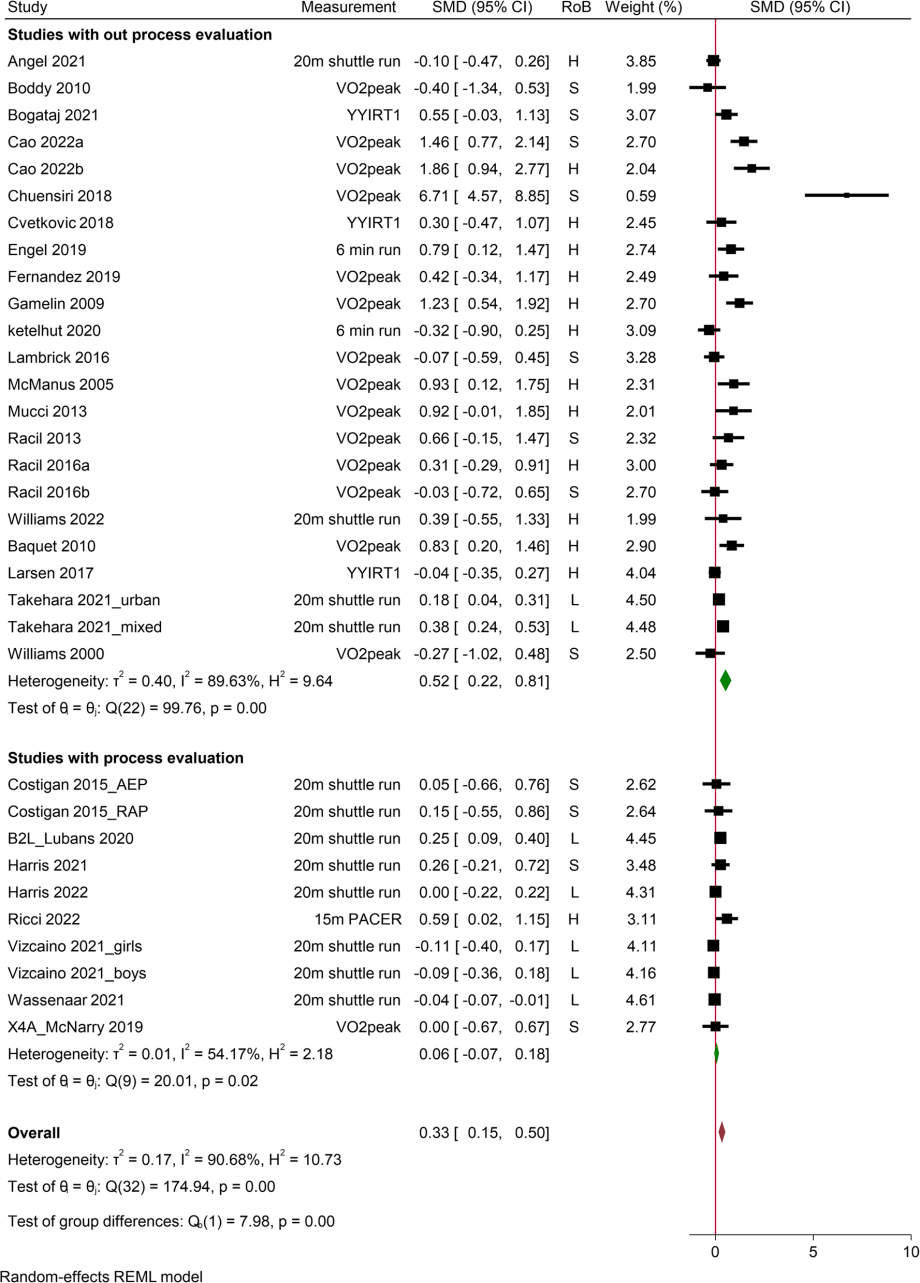
Studies with versus without process evaluation on CRF in school-based HIIT interventions. SMD, stand mean difference; RoB, risk of bias; CI, confidence interval; S, some concern; H, high risk of bias; L, low risk of bias; YYIRT1, YO-YO Intermittent Recovery Test Level 1; PACER, Progressive Aerobic Cardiovascular Endurance Run; AEP, aerobic exercise programme; RAP, resistance and aerobic programme

**Fig. 3:**
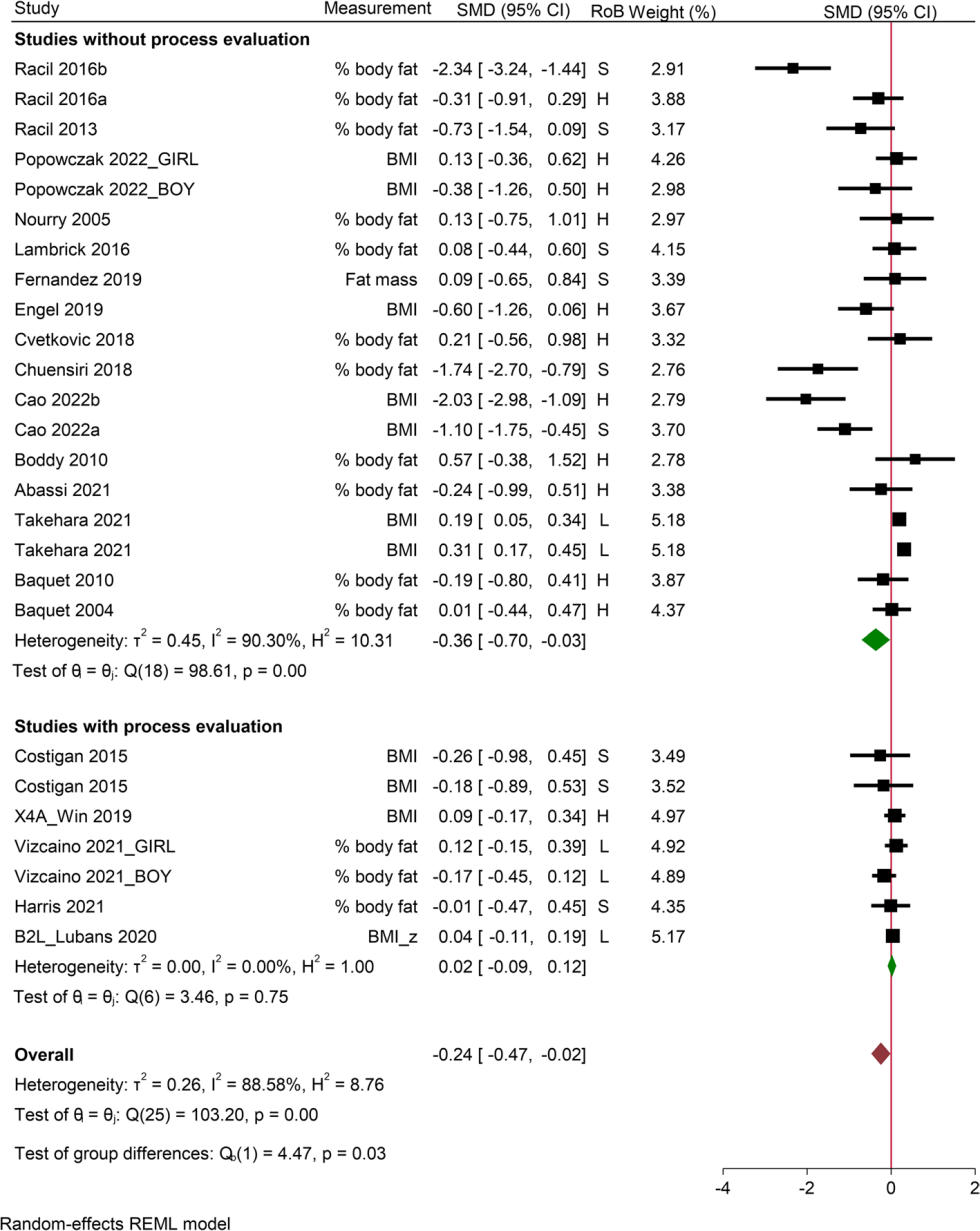
Studies with versus without process evaluation on body composition in school-based HIIT interventions. SMD, stand mean difference; RoB, risk of bias; CI, confidence interval; S, some concern; H, high risk of bias; L, low risk of bias; BMI, body mass index; AEP, aerobic exercise programme; RAP, resistance and aerobic programme

**Fig. 4:**
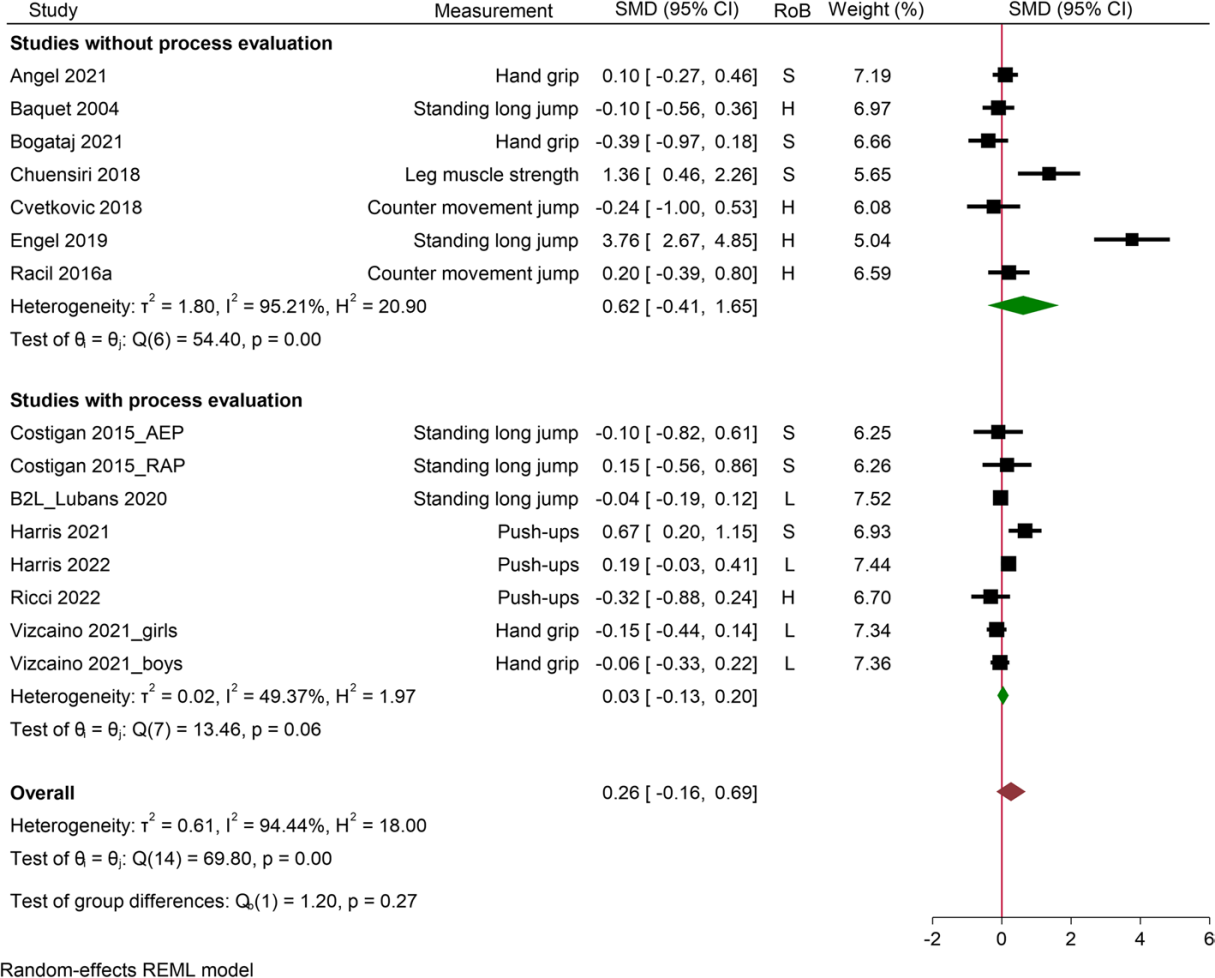
Studies with versus without process evaluation on muscular strength in school-based HIIT interventions. SMD, stand mean difference; RoB, risk of bias; CI, confidence interval; S, some concern; H, high risk of bias; L, low risk of bias; AEP, aerobic exercise programme; RAP, resistance and aerobic programme

No conflicts were found between the two authors regarding the risk of bias assessment of the 30% sub-sample. The details of risk of bias assessment for CRF, body composition and muscular strength are presented in Additional File 5 and 6, and are briefly displayed in Figs. 2, 3 and 4, respectively. Of the 30 studies reporting CRF, 5 (17%) studies were assessed as “low risk”, 11 (37%) “some concerns” and 14 (46%) “high risk”. For body composition outcome studies, 3 (14%) were rated as “low risk”, 8 (36%) “some concerns” and 11 (50%) “high risk”. For muscular strength outcome studies, 3 (23%) were assessed as “low risk”, 5 (38%) “some concerns” and 5 (39%) “high risk”. The major reasons for raising the concerns were: 1) lack of proper randomization; 2) no blinding; 3) not accounting for missing data; and 4) lack of pre-determined protocol.

### The extent of process evaluation reporting

Table 4 summaries the process evaluation measures across the included interventions. In total, 4 interventions labelled and nested process evaluation in a section of the paper [33–36] and one intervention reported process evaluation in a separate paper [37]. Although not labelled as “process evaluation”, two interventions published a separate paper to evaluate and reflect on the implementation process [60] and to identify the facilitators and barriers across the intervention delivery process [61]. On average, half of the process evaluation measures (*n* = 6) were reported upon, and most interventions (*n* = 43) reported on multiple process evaluation measures. Implementation was the most frequently reported domain (59%), followed by mechanism of impact (42%) and context (34%). The detailed information of how each process evaluation measures were met is presented in Table 5.

**Table 4:** Summary table for process evaluation measures across included interventions

Studies	Implementation process	Implementation					Mechanism of impact				Context			Summary
		Fidelity	Reach	Dose delivered	Recruitment & retention	Adaptation	Mediator	Dose Received	Unintended consequences	Responses	Barriers	Facilitators	Contamination	
Ariza et al. [49, 62]	N	Y	Y	Y	Y	N	Y	N	Y	N	N	Y	Y	8
McNarry et al. [51, 61, 63–66]	Y	Y	N	Y	Y	N	Y	N	Y	Y	Y	Y	Y	9
Williams et al. [67]	N	Y	N	Y	Y	Y	N	Y	Y	N	N	N	N	6
Martinez et al. [68]	N	N	N	Y	N	N	N	N	N	N	N	N	N	1
Lubans et al. [37, 50, 69–73]	Y	Y	Y	Y	Y	N	Y	Y	Y	Y	Y	Y	Y	11
Engel et al. [48]	N	Y	N	Y	Y	Y	N	Y	N	N	N	Y	N	6
Nourry et al. [74, 75]	N	Y	N	Y	Y	N	N	N	N	N	N	N	N	3
Baquet et al. [76]	N	Y	N	Y	Y	N	N	N	Y	N	N	N	N	4
Baquet et al. [77]	N	Y	Y	Y	Y	N	N	N	N	N	N	N	N	4
Angel et al. [78]	N	Y	Y	Y	Y	N	N	N	Y	N	N	Y	N	6
Chuensiri et al. [79]	N	Y	N	Y	Y	N	N	N	N	Y	N	N	N	4
Cardenosa et al. [80]	N	Y	N	Y	Y	N	N	N	N	N	N	N	N	3
Oliveira et al. [45]	N	Y	Y	Y	Y	N	N	Y	Y	N	N	N	Y	7
Racil et al. [81]	N	Y	N	Y	Y	N	N	N	N	N	N	N	N	3
Stenman et al. [82]	N	Y	N	Y	Y	N	N	N	N	N	N	N	N	3
Costigan et al. [33, 83, 84]	Y	Y	Y	Y	Y	N	Y	N	Y	Y	N	Y	Y	9
Harris et al. [36]	Y	Y	Y	Y	Y	Y	Y	Y	Y	Y	Y	Y	Y	12
Takehara et al. [53, 85]	N	N	Y	Y	Y	Y	Y	N	Y	N	N	Y	Y	8
Larsen et al. [86–88]	N	N	Y	Y	Y	Y	N	N	Y	Y	N	Y	Y	8
Moreau et al. [52]	N	N	N	Y	Y	N	N	N	N	N	Y	N	Y	4
Fernandez et al. [89]	N	Y	N	Y	N	N	N	N	N	N	N	N	N	2
McManus et al. [90]	N	Y	N	Y	Y	N	N	N	N	N	N	N	N	3
Racil et al. [91]	N	Y	N	Y	N	N	N	N	Y	N	N	N	N	3
Lambrick et al. [92, 93]	N	Y	N	Y	Y	N	Y	Y	Y	Y	N	Y	N	8
Vizcaino et al. [34, 94]	Y	Y	Y	Y	Y	Y	Y	Y	Y	Y	Y	Y	Y	12
Haghshenas et al. [95]	N	N	N	Y	N	N	N	N	N	N	N	N	N	1
Wassenaar et al. [43, 60, 96]	Y	Y	Y	Y	Y	Y	Y	Y	Y	Y	Y	Y	Y	12
Tian et al. [97]	N	Y	N	Y	Y	N	N	N	N	N	N	N	N	3
Popowczak et al. [55, 98–104]	N	N	N	Y	Y	N	Y	N	Y	N	N	N	N	4
Boddy et al. [105]	N	Y	N	Y	Y	Y	N	Y	N	N	Y	Y	N	7
McManus et al. [106]	N	N	N	Y	Y	N	N	Y	N	N	N	N	N	3
Amigo et al. [107, 108]	N	Y	N	Y	Y	N	Y	Y	Y	N	N	N	N	6
Cao et al. [109]	N	Y	Y	Y	Y	N	N	N	Y	N	N	N	Y	6
Cao et al. [110]	N	Y	Y	Y	Y	Y	Y	Y	Y	N	N	N	Y	9
Bogataj et al. [111]	N	Y	Y	Y	Y	N	N	Y	Y	N	Y	Y	Y	9
Gamelin et al. [112]	N	Y	N	Y	Y	N	N	N	N	N	N	N	N	3
Ketelhut et al. [113]	N	Y	N	Y	N	N	N	Y	Y	N	N	Y	N	5
Cvetković et al. [114, 115]	N	Y	Y	Y	Y	N	N	N	Y	N	Y	N	N	6
Abassi et al. [116, 117]	N	Y	N	Y	Y	N	N	N	Y	N	N	N	N	4
Racil et al. [118]	N	Y	N	Y	Y	N	N	Y	Y	N	N	N	N	5
Harris et al. [44]	Y	Y	Y	Y	Y	Y	Y	Y	Y	Y	Y	Y	Y	12
Ricci et al. [35]	Y	Y	Y	Y	Y	N	N	Y	Y	Y	Y	Y	N	9
Williams et al. [119]	N	Y	N	Y	Y	N	Y	Y	N	Y	N	Y	N	7
Logan et al. [47]	N	Y	Y	Y	Y	Y	Y	Y	Y	N	Y	N	N	9
Bossmann et al. [120]	N	Y	N	Y	Y	N	Y	Y	N	Y	N	Y	N	7

**Table 5:** How process evaluation measures were achieved across the included studies

Process evaluation measures	N	How	References
Fidelity	17	HR with data presented	[33, 34, 36, 37, 44, 45, 47, 49–51, 61–67, 69–73, 80, 82–84, 92–94, 105, 107, 108, 113, 119]
	5	HR without data presented	[79, 89, 90, 97, 111]
	6	MAS with description of how	[76, 77, 81, 109, 110, 114, 115]
	5	MAS without description of how	[74, 75, 91, 112, 116–118]
	2	RPE (mean 6.94/10 and 17.3/20, respectively)	[48, 78]
	2	RPE without data presented	[89, 120]
	2	VPA with one significantly different from control while the other not	[35], [43, 60, 96]
Reach	19	NA	[33–37, 43–45, 47, 49, 50, 52, 53, 60, 62, 69–73, 77, 78, 83–88, 94, 96, 109–111, 114, 115]
Dose delivered	45	NA	All included studies
Recruitment and retention	6	Dropped out due to lack of time	[36, 43, 60, 79, 82, 96, 110, 114, 115]
	6	Dropped out due to school transfer	[34, 36, 37, 44, 50, 53, 69–73, 85–88, 94]
	7	Excluded due to inability to reach requirement to be included	[49, 62, 74, 75, 78, 86–88, 91, 112, 116, 117]
	6	Dropped out due to illness	[45, 47, 76, 82, 111, 119]
	14	Excluded due to absence in testing days	[34, 36, 37, 44, 50, 52, 69–73, 77, 80, 86–88, 94, 97, 110, 111, 114–117, 120]
	6	Provided dropped out number without reasons	[33, 35, 51, 61, 63–67, 83, 84, 90, 106]
	4	100% compliance	[48, 105, 109, 118]
Adaptation	3	For intensity justification	[34, 94, 105, 110]
	5	For facilitating implementation	[36, 44, 48, 53, 85–88]
	3	For making a compromise	[43, 47, 60, 67, 96]
Mediator	6	Baseline level mediates outcomes	[37, 49, 50, 53, 55, 62, 69–73, 85, 92, 93, 98–104, 120]
	3	Baseline level did not mediate outcomes	[33, 43, 44, 60, 83, 84, 96]
	6	Sex	[33, 43, 44, 55, 60, 83, 84, 96, 98–104, 107, 108]
	3	Maturity	[36, 44, 51, 61, 63–66, 107, 108]
	3	Dose	[34, 43, 47, 60, 94, 96]
Dose received	20	Provided with how many doses are delivered	[34, 36, 37, 43, 44, 47, 48, 50, 60, 67, 69–73, 92–94, 96, 105–108, 110, 111, 113, 119, 120]
	3	100% compliance to the training sessions	[35, 45, 118]
Unintended consequences	22	Reported no adverse event	[33, 35–37, 45, 47, 49–51, 53, 55, 61–66, 69–73, 76, 78, 83–85, 91–93, 98–104, 107–111, 113–118]
	2	Minor injuries/dizziness	[86–88], [34, 94]
	1	One deliverer (teacher) absents from intervention delivery	[44]
	1	Extraordinary poor compliance and high dropout rate	[43, 60, 96]
	1	Inclement weather changed the outcome assessment plan	[67]
Response	12	Participants	[33–37, 44, 50, 51, 61, 63–66, 69–73, 79, 83, 84, 86–88, 92–94, 119, 120]
	6	Teachers	[33, 35–37, 43, 44, 50, 60, 69–73, 83, 84, 96]
	1	Parents	[34, 94]
	1	School authority	[43, 60, 96]
Barrier	3	Busy curriculum	[37, 43, 47, 50, 60, 69–73, 96]
	3	Inconvenient equipment	[35, 37, 50, 69–73, 105]
	6	Lack of time	[34, 36, 43, 51, 60, 61, 63–66, 94, 96, 111, 114, 115]
	3	Lack of space	[36, 43, 52, 60, 96]
	1	Lack of perceived improvement	[43, 60, 96]
Facilitator	12	Incentives	[33–36, 43, 51, 53, 60, 61, 63–66, 83–85, 92–94, 96, 105, 113, 119, 120]
	5	Theory models involved	[33, 35–37, 43, 50, 60, 69–73, 83, 84, 96]
	2	Study design	[48, 119]
	3	Support from research teams and schools	[37, 49, 50, 62, 69–73, 86–88]
	9	Pre-intervention training	[33, 37, 43, 44, 48, 50, 53, 60, 69–73, 78, 83–85, 96, 113, 119]
Contamination	3	Blinding of control group	[43, 49, 52, 60, 62, 96]
	11	Blinding of assessors or analyser	[33, 34, 36, 37, 44, 45, 50, 52, 53, 69–73, 83–85, 94, 109–111]
	1	Blinding of the randomisation process	[65, 105, 106]

*HR* heart rate, *MAS* maximum aerobic speed, *RPE* rating of perceived exertion, *VPA* vigorous physical activity, *NA* not applicable

#### Implementation 

In total, 38 out of 45 interventions (84%) reported fidelity using a variety of methods. Specifically, HR monitors were used in 22 interventions, with seventeen providing HR outcome data and five did not. Maximum aerobic speed (MAS) was adopted by eleven interventions. However, five of them did not articulate how MAS was used to quantify intensity (e.g., individualising interval distance according to participants’ MAS). Four interventions used rating of perceived exertion (RPE), with only two of them reporting outcome data (6.9/10 [78] and 17.3/20 [48]). Fidelity was also reported as accumulated VPA time via accelerometer by two interventions, with one demonstrating significantly higher VPA time in the intervention group compared to control [35]. Seven interventions did not monitor exercise intensity.

Reach was reported in 19 interventions (42%). All the interventions documented the number of participants recruited, despite five which did not provide information regarding retention. Of the 40 interventions that tracked retention, six recorded the number of dropped out participants but without specifying the reasons. Four interventions reported 100% attendance, while 31 reported both attritions and reasons, with lack of time, school transfer, illness and absence in testing days been most frequently documented. All the interventions provided information regarding dose delivered. Eleven interventions (22%) reported adaptation(s) to refine the implementation.

#### Mechanism of impact

While dose delivered was frequently reported, nearly half (22 interventions, 49%) did not track dose received. There were 16 interventions (36%) which investigated the mediators of the intervention outcomes, with baseline level, sex, maturity, and dose response explored. Adverse event (*n* = 24) was the most frequently reported unintended consequences. Apart from that, absence of deliverer (due to illness), poor compliance and inclement weather were documented. With regard to response, 12 interventions (27%) collected feedback from students, teachers, parents and/or school authorities, via questionnaires, interviews, focus groups and/or surveys.

#### Context

Barriers were reported in 12 interventions (27%). Among them, three interventions perceived the busy curriculum as a barrier, three mentioned the inconvenient use of equipment and six reported time constrains. Others were lack of space and perceived fitness improvement. By contrast, 20 interventions (44%) informed the facilitators of implementation. Of them, twelve interventions used different incentives to motivate participants, including offering choices (e.g., choose exercise modalities, partners or music), equipment (e.g., real-time HR on screen) and voucher/money upon completion of intervention; nine interventions provided pre-intervention training to students and/or teachers; five interventions adopted a theory model to guide the implementation; two interventions perceived their study design as facilitators, such as short, simple, low cost and equipment free; and three perceived the support from the schools or research team as facilitators. Lastly, 15 interventions (33%) reported the measurement of contamination. In detail, three interventions served the control groups with placebo (e.g., stretches); eleven blinded the outcome assessors to avoid bias in data collection; and one blinded the researchers for randomisation to ensure the quality of group allocation.

### Effects of process evaluation and intervention characteristics on CRF, body composition and muscular strength

A total of 33 studies were included for pooled random-effects meta-analyses and meta-regressions, with subgroup comparison between studies with and without process evaluation. In total, eight studies were appraised as fulfilling implementation process (intended to report process evaluation measures) and were allocated to the studies with process evaluation subgroup.

#### CRF

Thirty studies reported CRF related outcomes, including VO_2peak_ (*n* = 14), 20 m shuttle run (*n* = 13), Yo-Yo Intermittent Recovery Test Level 1 (*n* = 3), 6 min running (*n* = 2), and one study reported 15 m Progressive Aerobic Cardiovascular Endurance Run (Fig. 2). There was a small but significant overall improvement in CRF following HIIT compared to no exercise control group, with a SMD of 0.33 (95% CI 0.16 to 0.51; *I*^2^ = 90.90%; τ^2^ = 0.18). The subgroup analysis revealed a nonsignificant effect on CRF for studies with process evaluation (SMD 0.06; 95% CI -0.07 to 0.18; *I*^2^ = 54.18%; τ^2^ = 0.01), while a significant medium effect for studies without process evaluation (SMD 0.53; 95% CI 0.23 to 0.84; *I*^2^ = 89.87%; τ^2^ = 0.42) (Fig. 2).

Several sensitivity analyses were performed. By removing high risk of bias studies (*n* = 14), the overall effect for CRF remained significant (SMD 0.14; 95% CI 0.01 to 0.27; *I*^2^ = 77.19%; τ^2^ = 0.04). However, when accounting for risk of bias, both studies with and without process evaluation groups were not significant (SMD 0.03; 95% CI -0.09 to 0.15; *I*^2^ = 51.94%; τ^2^ = 0.01 and SMD 0.29; 95% CI -0.03 to 0.61; *I*^2^ = 83.98%; τ^2^ = 0.15, respectively). The removal of computed outcome scores [33, 35, 50, 53, 55] had no significant influence on the overall results. In addition, no individual study had a clinically or statistically meaningful effect on the overall SMD through leave-one-out analysis. The funnel plot indicated considerable asymmetry and Egger’s test (*p* < 0.01) showed significant publication bias for studies reporting CRF in school-based HIIT interventions. The sensitive analysis figures and funnel plot for CRF are presented in Additional File 7.

In total, 14 intervention characteristics and 15 process evaluation measures were regressed to examine mediators of CRF (Table 6). Altogether, seven intervention characteristics and four process evaluation measures significantly altered CRF. Specifically, the following characteristics elicited significantly greater CRF: 1) individual compared to cluster RCTs; 2) direct compared to indirect measurement of CRF; 3) overweight and/or obese cohort compared to not specified; 4) shorter compared to longer intervention duration; 5) running/cycling-based HIIT compared to other modalities; 6) higher compared to lower risk of bias studies; and 7) lower compared to higher reported % HR_max_. In addition, studies reported adaptation, dose received, incentive strategy and pre-intervention training were associated with significantly lower CRF compared to studies did not report these process evaluation measures (Table 6).

**Table 6:** Univariable meta-regressions for CRF in school-based high-intensity interval training interventions

Covariate of interest	n	β (SE)	*p* value	*I*^2^, %	*R*^2^, %
**Study characteristics**					
Randomisation (individual vs. cluster)	19 vs. 14	-0.45 (0.16)	**0.01**	86.96	31.03
Measurement (direct vs indirect)	13 vs. 20	-0.38 (0.19)	**0.05**	89.57	13.55
Age (years)	33	-0.01 (0.04)	0.86	90.83	0
Sex (Not specified vs. boys/girls)	20 vs. 13	0.11 (0.20)	0.57	91.4	0
Weight status (Not specified vs. overweight/obese)	25 vs. 8	0.66 (0.21)	**0.01**	86.32	37.31
Duration (weeks)	33	-0.02 (0.01)	**0.02**	78.52	36.5
Frequency (< 3 vs ≥ 3)	11 vs. 22	0.23 (0.12)	0.23	90.4	0
Session length (minutes)	33	-0.01 (0.01)	0.91	88.45	0
Work-to-rest ratio (< 1 vs ≥ 1)	5 vs. 24	-0.25 (0.31)	0.42	88.45	17.66
Deliverer (researcher vs. teacher)	19 vs. 9	-0.19 (0.22)	0.37	90.17	0
Modality (running/cycling vs. others)	13 vs. 20	-0.60 (0.17)	**0.01**	85.86	37.98
Occasion (physical education vs. others)	12 vs. 16	0.03 (0.20)	0.87	91.08	0
Risk of bias	33	0.21 (0.10)	**0.04**	88.45	17.66
**Process evaluation measures**					
Fidelity (no vs. yes)	4 vs. 29	0.23 (0.26)	0.37	89.17	0
HR value (%HRmax)	11	-0.01 (0.01)	**0.01**	0	100
Recruitment and retention (no vs. yes)	4 vs. 29	0.19 (0.31)	0.54	91.64	0
Reach (no vs. yes)	15 vs. 18	-0.13 (0.19)	0.49	91.27	0
Adaptation (no vs. yes)	17 vs. 16	-0.34 (0.17)	**0.05**	89.16	15.06
Mediator (no vs. yes)	19 vs. 14	-0.32 (0.17)	0.07	89.02	14.41
Dose received (no vs. yes)	17 vs. 16	-0.34 (0.18)	**0.05**	86.49	10.49
Response (no vs. yes)	22 vs. 11	-0.17 (0.19)	0.35	86.74	6.29
Adverse event (no vs. yes)	11 vs. 22	-0.09 (0.21)	0.95	84.92	0
Theoretical concept (no vs. yes)	27 vs. 6	-0.17 (0.23)	0.46	87.21	0
Incentive (no vs. yes)	20 vs. 13	-0.38 (0.18)	**0.03**	89.37	7.06
Training (no vs. yes)	19 vs. 14	-0.34 (0.17)	**0.05**	89.07	15.09
Barriers (no vs. yes)	22 vs. 11	-0.35 (0.18)	0.06	86.54	8.37
Contamination (no vs. yes)	18 vs. 15	-0.18 (0.18)	0.32	90.73	0
Overall process evaluation (≤ 7 vs. > 7)	17 vs. 16	-0.33 (0.17)	0.06	89.08	16.19

*SE* standard error

*p* values for significant covariates (*p* < 0.05) are highlighted in bold

#### Body composition

Data for body composition were available in 22 studies and five studies in the process evaluation subgroup (Fig. 3). Based on Table 2, data were presented as % body fat (*n* = 12), BMI (*n* = 8), BMI-z score (*n* = 1) and fat mass (*n* = 1). The pooled data showed significantly small effects on body composition in both overall (SMD = -0.24; 95% CI from -0.47 to -0.02; *I*^2^ = 88.58%; τ^2^ = 0.26) and studies without process evaluation group (SMD = -0.36; 95% CI from -0.70 to -0.03; *I*^2^ = 90.30%; τ^2^ = 0.45). By contrast, the studies with process evaluation showed no treatment effect (SMD = 0.02; 95% CI from -0.09 to 0.12; *I*^2^ = 0%; τ^2^ = 0).

When removing the high risk of bias studies (*n* = 11), neither overall nor studies with and without process evaluation subgroups were significant, whereas no significant change was observed by omitting the computed outcome scores. The results are listed in the Additional File 7. The funnel plot (Additional File 7) indicated slight asymmetry and Egger’s test (*p* = 0.09) suggested nonsignificant publication bias for studies measuring the outcome of body composition.

Meta-regression revealed that studies individually randomised, conducted among overweight and/or obese cohort, adopted running/cycling modality and without reporting incentive strategies induced significantly better effects on body composition compared to the counterparts (Table 7).

**Table 7:** Univariable meta-regressions for body composition in school-based high intensity interval training interventions

Covariate of interest	n	β (SE)	*p* value	*I*^2^, %	*R*^2^, %
**Study characteristics**					
Randomisation (individual vs. cluster)	15 vs. 7	0.51 (0.20)	**0.01**	83.38	35.46
Measurement (direct vs. indirect)	14 vs. 8	0.01 (0.24)	0.97	89.00	0
Age (years)	22	0.15 (0.30)	0.62	90.59	0
Sex (Not specified vs. boys/girls)	12 vs. 10	-0.39 (0.23)	0.48	87.24	8.76
Weight status (Not specified vs. overweight/obese)	14 vs. 8	-0.93 (0.17)	**0.01**	54.79	84.70
Duration (weeks)	22	0.01 (0.02)	0.78	88.50	0
Frequency (< 3 vs ≥ 3)	8 vs. 14	-0.40 (0.24)	0.10	87.86	13.59
Session length (minutes)	22	0.01 (0.01)	0.44	89.99	0
Work-to-rest ratio (< = 1 vs > 1)	13 vs. 7	0.46 (0.27)	0.08	83.29	9.12
Deliverer (researcher vs. teacher)	12 vs. 5	0.25 (0.32)	0.45	91.55	0
Modality (running/cycling vs. others)	10 vs. 12	0.69 (0.20)	**0.01**	79.68	50.11
Occasion (physical education vs. others)	6 vs. 11	0.07 (0.23)	0.75	84.79	0
Risk of bias	22	-0.09 (0.15)	0.52	88.48	0
**Process evaluation measures**					
Fidelity (no vs. yes)	4 vs. 18	0.14 (0.31)	0.64	89.45	0
HR value (%HRmax)	8	-0.01 (0.01)	0.83	0	0
Recruitment and retention (no vs. yes)	2 vs. 19	-0.14 (0.49)	0.77	89.60	0
Reach (no vs. yes)	13 vs. 9	-0.07 (0.26)	0.80	90.46	0
Adaptation (no vs. yes)	14 vs. 8	0.25 (0.24)	0.29	88.02	0
Mediator (no vs. yes)	14 vs. 8	0.38 (0.22)	0.08	86.22	19.94
Dose received (no vs. yes)	14 vs. 8	-0.18 (0.25)	0.47	87.95	0
Response (no vs. yes)	17 vs. 5	0.03 (0.27)	0.91	88.13	0
Adverse event (no vs. yes)	4 vs. 18	0.21 (0.32)	0.50	89.97	0
Theoretical concept (no vs. yes)	19 vs. 3	0.19 (0.32)	0.55	87.68	0
Incentive (no vs. yes)	15 vs. 7	0.53 (0.21)	**0.01**	84.31	28.48
Training (no vs. yes)	18 vs. 4	0.38 (0.24)	0.11	86.97	10.79
Barriers (no vs. yes)	16 vs. 6	0.35 (0.27)	0.19	88.62	0.11
Contamination (no vs. yes)	12 vs. 10	-0.02 (0.26)	0.93	90.75	0
Overall process evaluation (≤ 7 vs. > 7)	13 vs. 9	0.12 (0.25)	0.63	90.31	0

*SE* standard error

*p* values for significant covariates (*p* < 0.05) are highlighted in bold

#### Muscular strength

Thirteen studies reported muscular strength related variables, with 6 studies allocated to the process evaluation subgroup (Fig. 4). In terms of measurement, three studies reported handgrip, one for leg muscle strength, four for standing long jump, three for push-ups and two for counter movement jump. The overall effect was not significant, with SMD = 0.26 (-0.16, 0.69), *I*^2^ = 94.44% and τ^2^ = 0.61. Similarly, no significant findings were observed in the subgroup analyses (Fig. 4) and sensitive analyses (Additional File 7). The funnel plot (Additional File 7) indicated asymmetry and Egger’s test (*p* = 0.01) suggested significant publication bias for studies measuring the outcome of muscular strength. Furthermore, leave-one-out analysis did not modify the overall result. However, reporting of reach and adverse event were negatively associated with muscular strength outcomes (Table 8).

**Table 8:** Univariable meta-regressions for muscular strength in school-based high-intensity interval training interventions

Covariate of interest	n	β (SE)	*p* value	*I*^2^, %	*R*^2^, %
**Study characteristics**					
Randomisation (individual vs. cluster)	7 vs. 6	-0.42 (0.39)	0.28	92.77	0
Age (years)	13	-0.03 (0.08)	0.67	92.11	0
Sex (Not specified vs. boys/girls)	8 vs. 5	0.25 (0.41)	0.54	92.65	0
Weight status (Not specified vs. overweight/obese)	9 vs. 3	0.21 (0.52)	0.69	93.53	0
Duration (weeks)	13	-0.03 (0.02)	0.23	92.34	0
Frequency (< 3 vs ≥ 3)	4 vs. 9	0.22 (0.42)	0.59	92.68	0
Session length (minutes)	13	-0.01 (0.01)	0.71	92.72	0
Work-to-rest ratio (< = 1 vs > 1)	7 vs. 6	0.51 (0.40)	0.20	92.13	0
Deliverer (researcher vs. teacher)	6 vs. 6	0.59 (0.40)	0.14	92.27	7.49
Modality (running/cycling vs. others)	3 vs. 10	-0.06 (0.52)	0.91	93.59	0
Occasion (physical education vs. others)	5 vs. 6	0.40 (0.48)	0.40	94.30	0
Risk of bias	13	0.22 (0.25)	0.38	92.93	0
**Process evaluation measures**					
Fidelity (no vs. yes)	0 vs. 13	–	–	–	–
HR value (%HRmax)	5	0.01 (0.01)	0.74	69.77	0
Recruitment and retention (no vs. yes)	1 vs. 12	0.04 (0.81)	0.96	93.73	0
Reach (no vs. yes)	4 vs. 9	-0.96 (0.39)	**0.01**	90.22	28.14
Adaptation (no vs. yes)	7 vs. 6	0.29 (0.40)	0.47	93.16	0
Mediator (no vs. yes)	8 vs. 5	-0.28 (0.40)	0.47	93.18	0
Dose received (no vs. yes)	6 vs. 7	0.14 (0.41)	0.73	93.39	0
Response (no vs. yes)	7 vs. 6	0.29 (0.40)	0.47	93.16	0
Adverse event (no vs. yes)	1 vs. 12	-3.12 (0.54)	** < 0.01**	48.01	93.08
Theoretical concept (no vs. yes)	9 vs. 4	-0.23 (0.43)	0.59	91.98	0
Incentive (no vs. yes)	8 vs. 5	-0.41 (0.39)	0.29	92.05	0
Training (no vs. yes)	5 vs. 8	0.10 (0.42)	0.82	93.58	0
Barriers (no vs. yes)	6 vs. 7	-0.58 (0.37)	0.12	92.02	6.99
Contamination (no vs. yes)	7 vs. 6	-0.43 (0.39)	0.27	92.81	0
Overall process evaluation (≤ 7 vs. > 7)	6 vs. 7	-0.60 (0.38)	0.11	92.03	7.06

*SE* standard error

*p* values for significant covariates (*p* < 0.05) are highlighted in bold

## Discussion 

The present review is the first to scrutinise the extent of process evaluation reporting in school-based HIIT interventions while examining the influence of process evaluation and intervention characteristics on CRF, body composition, and muscular strength. Previous school-based HIIT reviews [18–23] exclusively focused on reporting intervention outcomes, overlooking the critical aspect of process evaluation. Our review timely addresses this gap by summarising the implementation process of included studies and determining the potential impact of process evaluation measures on intervention effectiveness for key outcomes including CRF, body composition and muscular strength.

### Summary of findings

In total, 77 studies from 45 school-based HIIT interventions were identified, with an average of 6 out of 12 process evaluation measures being reported. Five interventions (11%) explicitly labelled and reported process evaluation in either a section of the paper or summarised in a separate publication. However, most of them were atheoretical except one intervention [37] adopted a framework by McKay et al. [121]. Overall, half (6/12) of the process evaluation measures were reported on average across all the interventions, with implementation being the most frequently reported domain (59%), followed by mechanism of impact (42%) and context (34%). The current study did not identify any favourable associations between studies intended to report process evaluation and the intervention outcomes in terms of CRF, body composition and muscular strength, and neither did reporting any of the process evaluation measures elicit better treatment effects for these health parameters. Rather, the overall pooled studies and those studies without deliberately reporting process evaluation were found to have beneficial effects for CRF, body composition and muscular strength, despite the studies without process evaluation group being characterised by higher heterogeneity and risk of bias. These findings indicate that process evaluation elicits no salient potentiation to the intervention effectiveness. However, it is undeniable that process evaluation completes the outcome-oriented RCTs of school-based HIIT interventions by providing sound implementation details, exploring potential mechanisms of impact and clarifying context factors. Hence, the understanding of the intervention effectiveness, generalizability and transferability are enhanced through the “lens” of process evaluation. Nevertheless, process evaluation has been largely neglected and under-reported, which may have potentially tempered the value of the existing school-based HIIT interventions.

### How and to what extent process evaluation measures were reported and what can be used to inform future studies

Fidelity is the key to process evaluation and is defined as the degree of the intervention being delivered as intended by multiple frameworks [24, 121–123]. In the present review, it was represented solely by HIIT intensity. Of the 45 included interventions, the majority of studies (84%) monitored HIIT intensity. Notwithstanding, the prescribed intensity was monitored in various ways, and to some extent, in an incomplete or invalid manner. First, although HR monitors provide an objective measure of HIIT intensity, failing to report the actual HR data can compromise intervention fidelity and leave the mechanisms underlying intervention effectiveness ambiguous. Likewise, MAS is another way to prescribe intensity, however, not without the premise of articulating how to individualise the running speed. Second, RPE enables a simple and convenient way for intensity monitoring. Even so, notably, RPE is not yet validated in the context of school-based HIIT interventions. Interestingly, VPA (as measured via accelerometery) has emerged as a means of gaging HIIT intensity in two studies [35, 43], which ushered in a new direction and consideration for determining HIIT fidelity. Both studies aimed to assess intervention fidelity by comparing accelerometer determined VPA between the HIIT and control groups, with one study [35] reporting significantly greater amount of VPA time in the HIIT group, while the other [43] reported nonsignificant findings. However, neither study established a predetermined standard for ‘high intensity’ using VPA, despite fidelity being defined as intervention delivered as intended [24, 121–124]. Future studies are recommended to thoroughly consider the pros and cons of HIIT monitoring tools before incorporating into their study designs. These considerations should include factors such as precision, affordability, validity and time commitment. Additionally, the pursuit of more efficient tools for prescribing and monitoring HIIT could be a future research endeavour. Our research team has recently demonstrated the validity of utilising RPE, as evidenced by Liu et al. [125], and session RPE, as illustrated by Duncombe et al. [126], for monitoring HIIT in laboratory and school settings, respectively. Furthermore, a call is claimed to fully report the HIIT intensity data and to continuously working on developing convenient and feasible measurements for establishing HIIT fidelity, especially for large-scaled studies [18].

The current review found that the common reasons for dropping out were absence in testing days, illness, lack of time and school transfer. High level of attrition may lead to biased intervention effects [53], therefore, where possible, measures should be taken to motivate and retain participants or by using appropriate statistical analysis methods (e.g., intention-to-treat). Several strategies were applied to do so in the studies included in the present review, including providing a flexible intervention schedule [63], rescheduling the missed sessions or tests [47] and offering choices [35, 111] or rewards [36, 105, 119]. These are practical solutions for researchers to boost future “buy-in” of potential stakeholders. Since no “one size fits all” approach to the study design, adaptation(s) may be necessary at times. Yet, it was the least reported process evaluation measures. Findings suggested that the purposes for making the adaptation(s) were to: 1) adjust intensity (e.g., introduce new rules to avoid participants staying still [34]); 2) to ease implementation (e.g., substitute HR monitors with RPE for monitoring intensity [48]); and 3) to make a compromise (e.g., re-schedule sessions due to busy curriculum [47]). The current study demonstrated that HIIT is generally safe for children and adolescents in view of the fact that only minor injuries (bruises and strains) [86] and dizziness (due to blood sampling) [34] were reported. This was in accordance with previous reviews which demonstrated that HIIT is safe to be applied in children and adolescents [11, 127]. While dose delivered was reported by all the interventions, half of them (*n* = 23) were not clear on how many doses were received by participants, overshadowing the quality of implementation and the understanding of effectiveness. Although barriers and facilitators were underreported, the existing information sheds some light on future interventions. Based on the qualitative reporting of the included studies, it appears that busy curriculum [43, 50], lack of time [36, 111] and inconvenient equipment use [35, 105] were frequently reported as barriers to implementation, whereas training workshops [44, 48, 113], incentive strategies [105, 113] and theoretical instructions [37, 50] were reported as effective boosters. In addition, findings of the present review suggest that short, simple, enjoyable interventions that do not heavily rely on equipment may be better suited to meet the needs of stakeholders [48, 119].

### Effects of process evaluation measures

Nesting process evaluation within RCTs enables a comprehensive and lucid description of both the process and outcome evaluations, thereby facilitating the replication and synthesis of evidence [24]. Nevertheless, it remains unclear whether process evaluation leads to better effects in school-based HIIT interventions. The current review revealed that better reporting of process evaluation posed no potentiation to the outcomes of CRF, body composition and muscular strength in school-based HIIT interventions. Despite the context differences (e.g., setting, outcomes), findings in this review were contrary to those of previous studies. Seral-Cortes et al. [31] found that reporting of process evaluation measures was associated with significantly decreased BMI. Similarly, Ma et al. [30] claimed that the inclusion of a process evaluation aim tended to benefit the overall effectiveness of motor competence programmes.

Several explanations have been proposed for a better understanding of the counter intuitive findings. First, both studies with and without process evaluation were no longer significant with respect to CRF and body composition improvement after removing high risk of bias studies. This observation suggests that some of the high risk of bias studies might have distorted the overall effectiveness. Indeed, previous work has shown that higher risk of bias is associated with exaggerated (approximately 10%) treatment effects [128]. Given that 21/23 and 17/19 interventions in studies without process evaluation group for CRF and body composition respectively were appraised as either high risk or some concerns (see Figs. 2 and 3), there is a possibility that the effect size of these two outcomes in studies without process evaluation may have been overestimated.

Second, the studies included in this review had a disproportionately larger number of participants in the studies with process evaluation group (*n* = 8, participants = 17,774) compared to the studies without process evaluation group (*n* = 25, participants = 2,552), which may have contributed to the discrepancy in intervention effectiveness. Indeed, it is reported that when interventions are conducted at larger scales, they may experience a 'scale-up penalty' or 'voltage drop', where the effectiveness of the intervention diminishes due to adaptations made to accommodate the contexts [129, 130]. In agreement with this, the current review revealed that reporting of adaptation is associated with lower improvement in CRF (β = -0.34, *p* = 0.05) compared to studies that did not report adaptation. Third, studies with process evaluation group were mostly cluster-RCTs and utilised exercise modalities other than running/cycling, which were associated with lower improvements in CRF and body composition compared to the studies without process evaluation (Tables 6 and 7). It is, therefore, conceivable that process evaluation is unlikely the only reason, or not responsible, for intervention ineffectiveness. Fourth, it is worth considering that process evaluation may be independent of outcome evaluation if the intervention is conducted without a prescribed process evaluation aim and without the support of a process evaluation framework. This is supported by Ma et al. [30], who found that including a process evaluation aim tended to benefit the overall effectiveness of motor competence programmes. Thus, it seems plausible that process evaluation did not contribute to the outcome assessments in the current review since only one study [37] adopted a process evaluation framework. Nevertheless, future studies are encouraged to further explore the relationship between process and outcome evaluation.

### Effects of HIIT characteristics

The current review showed that studies conducted among overweight and obese cohort were associated with favourable intervention effects on CRF (*β* = 0.66, *p* = 0.01) and body composition (*β* = -0.93, *p* = 0.01) compared to studies without specifying weight status. This is in agreement with previous reviews [18, 22], which have demonstrated that overweight and obesity significantly mediates CRF, waist circumference, percentage body fat and BMI in school-based HIIT interventions targeting children and adolescents. Hence, HIIT may be a particular effective and beneficial form of exercise for this “at-risk” cohort [131, 132].

Previous school-based HIIT reviews have shown that running- or cycling-based HIIT was the most adopted modality [18, 20, 22]. Despite this, our review is the first to systematically compare the differences between the traditional running/cycling HIIT and other HIIT modalities (e.g., resistance-based HIIT). The pooled evidence suggested that running/cycling HIIT was superior in improving CRF and body composition, but not muscular strength, compared to other modalities. This finding corroborated the speculation made by Costigan et al. [11] that cycling/running-based HIIT was likely to improve CRF, rather than muscular strength, due to the lack of training specificity.

The current review found that shorter intervention duration elicited higher CRF improvement. This is consistent with Leahy et al. [133], where they concluded that shorter intervention duration emerged as a better predictor of well-being. In addition, Ma et al. [30] suggested that a shorter intervention duration led to greater effects on motor competence, proposing that longer interventions were more susceptible to interruptions and less supported. This assertion is supported by some of the studies included in the current review, indicating that extended interventions are associated with high drop-out rates and diminished implementation quality (e.g., inadequate exercise intensity monitoring and reduced dose delivery) [43, 53].

Interestingly, the pooled HR data revealed that lower HR was associated with significantly higher improvement in CRF, whereas no effects for body composition and muscular strength were observed. However, given the relatively low effect size (*β* = -0.01, *p* = 0.01), we speculate that HR induces no effects on the intervention outcomes once a certain level is reached (above 70% in the current review). This is supported by McKay et al. [134] and Schaun et al. [135] in which they stated that the magnitude of CRF would not be influenced by intensity once it is above 60% VO_2max_, despite their conclusion being based upon young adults.

### Strengths and limitations

This is the first review exploring the implementation process of school-based HIIT interventions in children and adolescents. The comprehensive literature search and combination of all possible outcomes regarding CRF, body composition and muscular strength contribute to the complete and overarching findings of the current school-based HIIT review. There are some limitations within this review. Since few studies had specified the process evaluation measures, most of the measures are subjectively assessed by authors. Although a second author in our review has checked for the accuracy and consistency of the judgements, disagreements may still exist from a reader’s point of view. To minimise author bias, the current review adopted the MRC process evaluation framework [24], combined with study by Ma et al. [30] for process evaluation. However, currently there is no single definition of process evaluation. The results may be different if another framework is adopted. Nevertheless, the MRC framework is probably one of the most comprehensive process evaluation guidance in the literature [136]. In addition, the preferential orders of data extraction for outcome variables in Table 1 is based on the understanding of authors in the present review. Consequently, it is essential to admit that these decisions may involve nuanced considerations. Finally, the results for all the outcomes showed significant methodological and statistical heterogeneity and publication bias, therefore future studies should interpret these results with caution.

## Conclusion

The extent of process evaluation in school-based HIIT interventions remains low, especially for the domains of adaptation, mediator, dose received, response, barriers, facilitators, and contamination. Even when interventions were conducted with the purpose of process evaluation, they generally lacked theoretical rigour. The present review suggests that process evaluation was not related to outcome evaluation and did not contribute to achieving better treatment effects. However, incorporating process evaluation into RCTs may be beneficial in providing comprehensive implementation details, which aids in interpreting intervention effectiveness and functioning. Ultimately, this will contribute to the scaling up and translation to other school-based HIIT interventions. Therefore, future school-based HIIT interventions are highly recommended to report process evaluation under the guidance of a theoretical framework.

### Adaptations from protocol

The sentence “cardiorespiratory fitness (20 m shuttle run in laps finished), body composition (BMI), strength (push-ups in times and standing long jump in meters) and blood pressure (mmHg)**”** in the protocol was changed to** “**cardiorespiratory fitness (e.g., 20 m shuttle run in laps finished), body composition (e.g., BMI), strength (e.g., push-ups in times and standing long jump in meters) and blood pressure (mmHg)**”.** In addition, the initial process of risk of bias assessments, conducted by two independent authors (YL and CW), was modified. The first author (YL) performed the risk of bias assessment, and the accuracy of the assessment was subsequently verified by a second author (CW) through a random sub-sample (30%) of studies, with less than 80% of consensus triggered a check for all the studies.

### Supplementary Information

**Additional file 1: Table S1. **PRISMA 2020 Checklist.

**Additional file 2. **

**Additional file 3:** **Table S2.** Exclusion details for the initial search and updated search.

**Additional file 4:** **Table S3.** Study characteristics.

**Additional file 5:** **Fig S1.** Risk of bias assessment for cardiorespiratory fitness. **Fig S2.** Risk of bias assessment for body composition. **Fig S3.** Risk of bias assessment for muscular fitness.

**Additional file 6. **

**Additional file 7: Fig S4.** Sensitive analysis by removing high risk of bias studies for CRF. N, participant number; SD, standard deviation; SMD, standard mean difference; CI, confidence interval; AEP, aerobic exercise programme; RAP, resistance and aerobic programme. **Fig S5.** Sensitive analysis by removing studies with computed outcome scores for cardiorespiratory fitness. N, participant number; SD, standard deviation; SMD, standard mean difference; CI, confidence interval. **Fig S6.** Funnel plot for studies reported cardiorespiratory fitness. **Fig S7.** Sensitive analysis by removing high risk of bias studies for body composition. N, participant number; SD, standard deviation; SMD, standard mean difference; CI, confidence interval; AEP, aerobic exercise programme; RAP, resistance and aerobic programme. **Fig S8.** Sensitive analysis by removing studies with computed outcome scores for body composition. N, participant number; SD, standard deviation; SMD, standard mean difference; CI, confidence interval. **Fig S9.** Funnel plot for studies reported body composition. **Fig S10.** Sensitive analysis by removing high risk of bias studies for muscular strength. N, participant number; SD, standard deviation; SMD, standard mean difference; CI, confidence interval; AEP, aerobic exercise programme; RAP, resistance and aerobic programme. **Fig S11.** Sensitive analysis by removing studies with computed outcome scores for muscular strength. N, participant number; SD, standard deviation; SMD, standard mean difference; CI, confidence interval. **Fig S12.** Funnel plot for studies reported muscular strength.

## Data Availability

All data generated or analysed during this study are included in this article.

## Abbreviations

HIIT: High-intensity interval training
CRF: Cardiorespiratory fitness
PA: Physical activity
MVPA: Moderate-to-vigorous physical activity
VPA: Vigorous physical activity
RCT: Randomised controlled trial
HRmax: Maximum heart rate
VO2peak: Peak oxygen uptake
BMI: Body mass index
SD: Standard deviation
SMD: Standard mean difference
MAS: Maximum aerobic fitness
RPE: Rating of perceived exertion
