# Supplementary material for: Process evaluation of school-based high-intensity interval training interventions for children and adolescents: a systematic review and meta-analysis of randomized controlled trials
Source: BMC Public Health. 2024 Feb 2;24:348. doi: 10.1186/s12889-024-17786-6 (PMC10835840; doi:10.1186/s12889-024-17786-6)
Supplement: Supplementary file 2 — Additional file 2. [file 12889_2024_17786_MOESM2_ESM.docx]

**Medline (Ovid)**

1 High-Intensity Interval Training/

2 (“high intensity” adj5 (interval? or intermittent or repeat* or circuit* or train* or exercis* or intervention?)).tw,kw.

3 (aerobic adj4 (interval? or intermittent or repeat* or circuit*)).tw,kw.

4 (sprint adj4 (interval? or intermittent or repeat* or circuit* or train* or exercis* or intervention?)).tw,kw.

5 (HIIT or HIIE).tw,kw.

6 Or/1-5

7 exp Child/

8 Child*.mp.

9 exp Adolescent/

10 Adolescen*.mp.

11 (youth? or teen* or boy? or girl?).mp.

12 student?.mp.

13 or/7-12

14 exp Schools/

15 school?.mp.

16 (break? or recess*2).mp.

17 class*2.mp.

18 "physical education".mp.

19 or/14-18

20 and/6,13,19

21 limit 20 to (english language and humans)

**SPORTDiscus (EBSCOhost)**

S1 SU interval training

S2 TI “high intensity” N4 (interval# or intermittent or repeat* or circuit* or train* or exercis* or intervention#)

S3 AB “high intensity” N4 (interval# orintermittent orrepeat* or circuit* or train* or exercis* or intervention#)

S4 TI Aerobic N3 (interval# or intermittent or repeat* or circuit*)

S5 AB Aerobic N3 (interval# or intermittent or repeat* or circuit*)

S6 TI sprint N3 (interval# or intermittent or repeat* or circuit* or train* or exercis* or intervention#)

S7 AB sprint N3 (interval# or intermittent or repeat* or circuit* or train* or exercis* or intervention#)

S8 TI HIIT or HIIE

S9 AB HIIT or HIIE

S10 S1 OR S2 OR S3 OR S4 OR S5 OR S6 OR S7 OR S8 OR S9

S11 TI child* or adolescen* or youth# or teen* or student# or boy# or girl#

S12 AB child* or adolescen* or youth# or teen* or student# or boy# or girl#

S13 S11 OR S12

S14 TX school# or "physical education" or break* or recess* or class*

S15 S10 AND S13 AND S14

S16 further limited to academic journals and English language

**Web of science**

1 TS=(interval training)

2 TI=(“high intensity” Near/4 (I nterval$ or intermittent or repeat* or circuit* or train* or exercis* or intervention$) )

3 AB=(“high intensity” Near/4 (interval$ or intermittent or repeat* or circuit* or train* or exercis* or intervention$) )

4 TI=(aerobic Near/3 (interval$ or intermittent or repeat* or circuit*) )

5 AB=(aerobic Near/3 (interval$ or intermittent or repeat* or circuit*) )

6 TI=(Sprint Near/3 (interval$ or intermittent or repeat* or circuit* or train* or exercis* or intervention$) )

7 AB=(Sprint Near/4 (interval$ or intermittent or repeat* or circuit* or train* or exercis* or intervention$) )

8 TI=(HIIT or HIIE)

9 AB=(HIIT or HIIE)

10 #1 OR #2 OR #3 OR #4 OR #5 OR #6 OR #7 OR #8 OR #9

11 TI=(child* or adolescen* or youth$ or teen* or student$ or boy$ or girl$ )

12 AB=(child* or adolescen* or youth$ or teen* or student$ or boy$ or girl$)

13 #11 OR #12

14 TS=(school$ or "physical education" or break$ or recess or recesses or class or classes)

15 #10 AND #13 AND #14

Document type: articles, languages: English

**SCOPUS**

(TITLE-ABS("high intensity" w/4 (interval# or intermittent or repeat* or circuit* or train* or exercis* or intervention#)) OR TITLE-ABS(aerobic w/3 (interval# or intermittent or repeat* or circuit*) ) OR TITLE-ABS(Sprint w/3 (interval# or intermittent or repeat* or circuit* or train* or exercis* or intervention#) ) OR TITLE-ABS(HIIT or HIIE )) AND (TITLE-ABS(child* or adolescen* or youth# or teen* or student# or boy# or girl#)) AND (ALL(school# or "physical education" or break# or recess or recesses or class or classes)) AND ( LIMIT-TO ( DOCTYPE,"ar" ) ) AND ( LIMIT-TO ( LANGUAGE,"English" ) )

**Cochrane Central Register of Controlled Trials**

#1 [mh ^"high intensity interval training"]

#2 “high intensity” Near/4 (interval? or intermittent or repeat* or circuit* or train* or exercis* or intervention?):ti,ab,kw

#3 aerobic Near/3 (interval? or intermittent or repeat* or circuit*):ti,ab,kw

#4 Sprint Near/3 (interval? or intermittent or repeat* or circuit* or train* or exercis* or intervention?):ti,ab,kw

#5 (HIIT or HIIE ):ti,ab,kw

#6 {OR #1-#5}

#7 (child* or adolescen* or youth? or teen* or student? or boy? or girl? ):ti,ab,kw

#8 school? or "physical education" or break? or recess or recesses or class or classes

#9 {AND #6-#8}
