# Supplementary material for: Process evaluation of school-based high-intensity interval training interventions for children and adolescents: a systematic review and meta-analysis of randomized controlled trials
Source: BMC Public Health. 2024 Feb 2;24:348. doi: 10.1186/s12889-024-17786-6 (PMC10835840; doi:10.1186/s12889-024-17786-6)
Supplement: Supplementary file 3 — Additional file 3: Table S2. Exclusion details for the initial search and updated search. [file 12889_2024_17786_MOESM3_ESM.docx]

Table S2. Exclusion details for the initial search and updated search

| Initial Search | | Updated search | |
| --- | --- | --- | --- |
| Excluded Studies | Reason | Excluded Studies | Reason |
| Barbeau 2003 | Excluded due to no reply | Amigo 2022 | Conference abstract |
| Gutin 2002 | Excluded due to no reply | Leite 2022 | Duplicate |
| Kang 2002 | Excluded due to no reply | Domaradzki 2022 | Duplicate |
| Kargarfard 2016 | Excluded due to no reply | González-Ruíz 2022 | Duplicate |
| Paahoo 2020 | Excluded due to no reply | Abassi 2022 | Duplicate |
| Paahoo 2021 | Excluded due to no reply | Julian 2022 | Protocols |
| Buchan 2011 | Excluded due to no reply | Juric 2022 | Protocols |
| Ellyas 2021 | Excluded due to no reply | Kable 2022 | Wrong population |
| Farpour-Lambert 2019 | Excluded due to no reply | Devereux 2022 | Wrong population |
| Segovia 2020 | Excluded due to no reply | Mendonça 2022 | Wrong population |
| Mucci 2013 | Excluded due to no reply | Chang 2022 | Wrong study design |
| Nikbakht 2012 | Excluded due to no reply | Wang 2022 | Wrong study design |
| Anderson 2018 | Conference abstract | Julian 2022 | Wrong study design |
| Anderson 2019 | Conference abstract | Nambi 2022 | Wrong study design |
| Ingul 2018 | Conference abstract | Aparecido 2022 | Wrong study design |
| Jprn 2018 | Conference abstract | MacLennan 2022 | Wrong study design |
| Leahy 2018 | Conference abstract | Gavanda 2022 | Wrong study design |
| Maud 2017 | Conference abstract | Lohonathan 2022 | Wrong population |
| Richmond 2011 | Conference abstract | Marinho 2022 | Wrong study design |
| Ricci 2021 | Conference abstract | Sick 2022 | Wrong study design |
| Kappenstein 2015 | Conference abstract | Androzzi 2022 | Wrong study design |
| McGavock 2014 | Conference abstract | Xiao 2022 | Wrong study design |
| Anderson 2019 | Conference abstract | Lu 2022 | Wrong study design |
| McKee 2021 | Conference abstract | Zhang 2022 | Wrong study design |
| Basuodan 2022 | Conference abstract | Leal 2022 | Wrong study design |
| McKee 2021 | Conference abstract | Colpitts 2022 | Wrong study design |
| Domaradzki 2020 | Duplicate | Faria 2022 | Wrong study design |
| Cvetković 2018 | Duplicate | Yang 2022 | Wrong study design |
| Engel 2019 | Duplicate | Zoellner 2022 | Wrong study design |
| Costigan 2018 | Duplicate | Salus 2022 | Wrong study design |
| Lambrick 2016 | Duplicate | Bouamra 2022 | Wrong study design |
| Martin-Smith 2019 | Duplicate |  |  |
| Anderson 2018 | Protocols |  |  |
| Beato 2022 | Protocols |  |  |
| Paulino da Silva Bento 2021 | Protocols |  |  |
| Cadenas-Sánchez 2016 | Protocols |  |  |
| Hu 2022 | Protocols |  |  |
| Thomas 2021 | Protocols |  |  |
| Kannan 2021 | Protocols |  |  |
| Duncombe 2022 | Protocols |  |  |
| Burford 2021 | Wrong study design |  |  |
| Faria 2020 | Wrong study design |  |  |
| Ahmadi 2020 | Wrong study design and population | |  |
| Alizadeh 2019 | Wrong population |  |  |
| Baquet 2002 | Wrong study design |  |  |
| Baquet 2017 | Wrong study design |  |  |
| Benson 2008 | Wrong study design |  |  |
| Burns 2012 | Wrong study design |  |  |
| Ingul 2017 | Wrong study design |  |  |
| Cooper 2018 | Wrong study design |  |  |
| Corte de Araujo 2012 | Wrong study design |  |  |
| de Lira 2017 | Wrong study design |  |  |
| Delgado-Floody 2018 | Wrong study design |  |  |
| Depiazzi 2021 | Wrong study design |  |  |
| Dias 2016 | Wrong study design |  |  |
| Doyle-Baker 2011 | Wrong study design |  |  |
| Fakhri 2020 | Wrong population |  |  |
| Farah 2014 | Wrong study design |  |  |
| Fidelix 2019 | Wrong study design |  |  |
| Granacher 2011 | Wrong study design |  |  |
| Hatch 2021 | Wrong study design |  |  |
| Jeon 2017 | Wrong study design |  |  |
| Juhanna 2018 | Wrong population |  |  |
| Julian 2021 | Wrong study design |  |  |
| Ma 2015 | Wrong study design |  |  |
| Martino 2022 | Wrong population |  |  |
| Miguet 2019 | Wrong study design |  |  |
| Morris 2018 | Wrong study design |  |  |
| Tottori 2019 | Wrong study design |  |  |
| Van Biljon 2018 | Wrong study design |  |  |
| Vakili 2021 | Wrong study design |  |  |
| Miguel 2019 | Wrong study design |  |  |
| McBride 2000 | Wrong study design |  |  |
| Heemskerk 2020 | Wrong study design |  |  |
| Rotstein 1986 | Wrong study design |  |  |
| Weston 2016 | Wrong study design |  |  |
| Ben-Zeev 2020 | Wrong study design |  |  |
| Chen 2021 | Wrong study design |  |  |
| De Fátima Aguiar Lopes 2021 | Wrong study design |  |  |
| Khammassi 2021 | Wrong study design |  |  |
| Malarvizhi 2021 | Wrong study design |  |  |
| Segovia 2020 | Wrong study design |  |  |
| Videira-Silva 2021 | Wrong study design |  |  |
| Weston 2021 | Wrong study design |  |  |
| Travlos 2010 | Wrong study design |  |  |
| Zakas 1994 | Wrong study design |  |  |
| Muntaner-Mas 2017 | Wrong study design |  |  |
| Jeon 2017 | Wrong study design |  |  |
| Prado 2015 | Wrong study design |  |  |
| Rosenkranz 2012 | Wrong study design |  |  |
| Tenório 2018 | Wrong study design |  |  |
| Tjønna 2009 | Wrong study design |  |  |
| Assuncao 2016 | Wrong study design |  |  |
| Cao 2012 | Wrong study design |  |  |
| Davis 2011 | Wrong study design |  |  |
| Esk 2020 | Wrong study design |  |  |
| Fern 2017 | Wrong study design |  |  |
| Farpour-Lambert 2019 | Wrong study design |  |  |
| Norris 1992 | Wrong study design |  |  |
| Owens 1999 | Wrong study design |  |  |
| Lira 2017 | Wrong study design |  |  |
| Dias 2018 | Wrong study design |  |  |
| Alemayehu 2018 | Wrong study design |  |  |
| Alvarez 2017 | Wrong study design |  |  |
| Lee 2021 | Wrong study design |  |  |
| Plavsic 2020 | Wrong population |  |  |
| Foster 2015 | Wrong population |  |  |
| Khammassi 2018 | Wrong population |  |  |
| Katsik Adelis 2017 | Wrong population |  |  |
| Mahfudz 2019 | Wrong population |  |  |
| Chia 2002 | Wrong population |  |  |
| Ko 2021 | Wrong population |  |  |
| Iraji 2020 | Wrong population |  |  |
| Gencay 2019 | Wrong population |  |  |
| Anderson 2021 | Wrong study design |  |  |
| Araujo 2012 | Wrong study design |  |  |
| Dias 2018 | Wrong study design |  |  |
| Thackray 2013 | Wrong study design |  |  |
| Lazzer 2011 | Wrong study design |  |  |
| Barker 2014 | Wrong study design |  |  |
| Wymbs 2021 | Wrong study design |  |  |
| Akhoundnia 2019 | Wrong study design |  |  |
| Bond 2015 | Wrong study design |  |  |
| Lazzer 2017 | Wrong study design |  |  |
| Chiwaridzo 2019 | Wrong study design |  |  |
| Ansell 2020 | Wrong population |  |  |
| Aschendorf 2019 | Wrong study design |  |  |
| Atashak 2018 | Wrong study design |  |  |
| Bang-Kittilsen 2021 | Wrong study design |  |  |
| Biwer 2003 | Wrong study design |  |  |
| Dorneles 2016 | Wrong study design |  |  |
| Engh 2015 | Wrong study design |  |  |
| Ferrete 2014 | Wrong population |  |  |
| Jayo-Montoya 2017 | Wrong study design |  |  |
| Kendall 2020 | Wrong study design |  |  |
| McBride 1998 | Wrong study design |  |  |
| Okamoto 2011 | Wrong study design |  |  |
| Rolid 2020 | Wrong study design |  |  |
| Schranz 2017 | Wrong study design |  |  |
| Tucker 2018 | Wrong study design |  |  |
| Biljon 2018 | Wrong study design |  |  |
| Zucchetti 2020 | Wrong study design |  |  |
| Benson 2007 | Wrong study design |  |  |
| Dehghani 2016 | Wrong study design |  |  |
| Eiholzer 2010 | Wrong study design |  |  |
| Gifari 2021 | Wrong study design |  |  |
| Lau 2015 | Wrong study design |  |  |
| Mattern-Baxter 2019 | Wrong study design |  |  |
| Schmidt 2016 | Wrong study design |  |  |
| Schmitz 2019 | Wrong study design |  |  |
| Yu 1997 | Wrong study design |  |  |
| Burley 2020 | Wrong study design |  |  |
| Cooper 2016 | Wrong study design |  |  |
| Duval 2017 | Wrong study design |  |  |
| Kucab 2021 | Wrong study design |  |  |
| Stepto 2012 | Wrong study design |  |  |
| Tabata 1990 | Wrong study design |  |  |
| Zwinkels 2019 | Wrong study design |  |  |
| Anjana 2021 | Wrong study design |  |  |
| McNarry 2020 | Wrong study design |  |  |
| Cockcroft 2015 | Wrong study design |  |  |
| Cooper 2016 | Wrong study design |  |  |
| Guedin 2019 | Wrong study design |  |  |
| Kohrt 2004 | Wrong study design |  |  |
| Lauglo 2016 | Wrong study design |  |  |
| Mullane 2017 | Wrong study design |  |  |
| Ouerghi 2017 | Wrong study design |  |  |
| Edge 2005 | Wrong study design |  |  |
| Mang 2016 | Wrong study design |  |  |
| Gavanda 2022 | Wrong study design |  |  |
| Devereux 2022 | Wrong study design |  |  |
| Ouerghi | Wrong population |  |  |
| Harris 2022 | Wrong study design |  |  |
| Zakas 2001 | Wrong study design |  |  |
| Brisebois 2021 | Wrong study design |  |  |
| Gerber 2014 | Wrong population |  |  |
| Hovanloo 2013 | Wrong study design |  |  |
| Kikuchi 2016 | Wrong study design |  |  |
