# Supplementary material for: Process evaluation of school-based high-intensity interval training interventions for children and adolescents: a systematic review and meta-analysis of randomized controlled trials
Source: BMC Public Health. 2024 Feb 2;24:348. doi: 10.1186/s12889-024-17786-6 (PMC10835840; doi:10.1186/s12889-024-17786-6)
Supplement: Supplementary file 4 — Additional file 4: Table S3. Study characteristics. [file 12889_2024_17786_MOESM4_ESM.docx]

Table S3. Study characteristics.

| Studies | Country | Randomization | N | Sex | Age (years) | Weight status | Duration (weeks) | Frequency | Work bout (s) | Rest bout (s) | Session length | Modality | Control group |
| --- | --- | --- | --- | --- | --- | --- | --- | --- | --- | --- | --- | --- | --- |
| Abassi et al. 2020, 2021 | Tunisia | RCT | 43 | girls | 16.7 | Overweight  /Obese | 12 | 3 | 30 | 30 | 20 | Running | Maintain daily life |
| Amigo et al. 2020, 2021 | Chile | RCT | 48 | mixed | 9.48 | NI | 11 | 2 | 240 | 120 | 24 | Games | Moderate-intensity games |
| Lopez 2018, Ariza 2019 | Spain | RCT | 184 | mixed | 13.7 | NI | 12 | 2 | 20, 25, 30, 35, 40 | 20, 25, 30, 35, 40 | 16 | Circuit exercises | Static stretching |
| Kennedy et al. 2020, Leahy et al. 2019a&b, Leahy et al. 2020, Lubans et al. 2020, Mavilidi et al. 2021 | Australia | Cluster-RCT | 670 | mixed | 16 | NI | 52 | 2 | 30 | 30 | 15 | Aerobic, Resistance, Dance, Boxing | Regular PE |
| Costigan et al. 2015&2016&2018 | Australia | RCT | 65 | mixed | 15.6 | NI | 8 | 3 | 30 | 30 | 9 | Resistance training | Regular PE |
| Cvetkovic et al. 2018a&b | Serbia | RCT | 42 | boys | 11 to 13 | Overweight  /Obese | 12 | 3 | 10, 15,20 | 10, 15,20 | 18 | Running | Regular PE |
| Popowczak et al. 2022, Domaradzki et al. 2020,2021, 2022a&b&c&d&e | Poland | Cluster-RCT | 141 | mixed | 16.2 | NI | 10 | 1 | 20 | 10 | 14 | Tabata | Regular PE |
| McNarry et al. 2015, Lambrick et al. 2016 | United Kingdom | RCT | 55 | mixed | 9.2 | Normal weight and obese | 6 | 2 | 360 | 120 | 40 | Games | Usual care |
| Elbe et al. 2016, Larsen et al. 2017, 2018 | Denmark | Cluster-RCT | 300 | mixed | 9.3 | NI | 44 | 3 | 60 | 30 | 40 | Games | Regular PE |
| Mucci et al. 2013, Nourry et al. 2005 | France | RCT | 18 | mixed | 10 | NI | 8 | 2 | 10 to 30 | 10 to 30 | 30 | Running | Usual care |
| Takehara et al. 2019, 2021 | Mongolia | Cluster-RCT | 1878 | mixed | 9.7 | NI | 10 | 2 | NI | NI | 10 to 25 | Running + resistance | Regular PE |
| Vizcaino et al. 2019, 2021 | Spain | Cluster-RCT | 487 | mixed | 10 | NI | 32 | 4 | 240 | 180 | 28 | Games | Regular PE |
| Wassenaar et al. 2019, 2020, 2021 | United Kingdom | Cluster-RCT | 18261 | mixed | 12 to 13 | NI | 52 | 2 | NI | NI | 10 | Squats, lunges | Regular PE |
| McNarry et al. 2019, 2020, 2021, Winn et al. 2018, 2021, Sharp et al. 2020 | United Kingdom | Cluster-RCT | 616 | mixed | 13 | NI | 24 | 3 | 10 or 30 | 10 or 30 | 30 | Circuits and games | Maintain daily life |
| Angel et al. 2021 | Denmark | RCT | 114 | mixed | 8 to 12 | NI | 10 | 3 | 240 | 120 | 30 | Games | Maintain daily life |
| Baquet et al. 2004 | France | Cluster-RCT | 53 | mixed | 9.7 | NI | 7 | 2 | 10 or 20 | 10 or 20 | 30 | Running | Regular PE |
| Baquet et al. 2010 | France | Cluster-RCT | 72 | mixed | 9.8 | NI | 7 | 3 | 10 to 30 | 10 to 30 | 6 to 20 | Running | Regular PE |
| Boddy et al. 2010 | United Kingdom | RCT | 16 | girls | 11.8 | NI | 3 | 4 | 30 | 45 | 36 | Dance | NI |
| Bogataj et al. 2021 | Serbia | RCT | 48 | girls | 15.5 | Overweight  /Obese | 8 | 3 | 30 | 15 | 15 | Resistance training | Regular PE |
| Cardenosa et al. 2016 | Spain | RCT | 35 | mixed | 11.2 | NI | 8 | 3 | 20 | 60, 40 or 20 | 10 | Running, sprint | MICT |
| Cheunsiri et al. 2018 | Thailand | RCT | 48 | boys | 11 | Overweight  /Obese | 12 | 3 | 120 | 60 | 24 | Cycling | Maintain daily life |
| Engel et al. 2019 | Germany | RCT | 35 | mixed | 11.7 | NI | 4 | 4 | 20,30,45,50 | 20,30,45,50 | 4 | Functional training | Maintain daily life |
| Fernandez et al. 2019 | Spain | RCT | 26 | mixed | 15 to 16 | NI | 7 | 2 | 20 | 20 | 4 to 8 | Resistance training | Regular PE |
| Gamelin et al. 2009 | France | RCT | 38 | mixed | 9.6 | NI | 7 | 3 | 10 to 30 | 10 to 30 | 23 | Running | NI |
| Haghshenas et al. 2019 | Iran | Cluster-RCT | 100 | boys | 12 to 16 | Overweight  /Obese | 8 | 3 | 240 | 180 | 25 to 43 | Running | Active walk |
| Harris 2021 | New Zealand | Cluster-RCT | 84 | mixed | 11.9 | NI | 8 | 2 | varied | varied | 15 | NI | Regular PE |
| Ketelhut et al. 2020 | Germany | Cluster-RCT | 46 | mixed | 10.8 | NI | 12 | 2 | 20 to 120 | 30 to 90 | 20 | Games, circuits | Regular PE |
| Logan et al. 2016 | New Zealand | RCT | 26 | boys | 16 | NI | 8 | 3 | 20 | 10 | 2 to 9 | Aerobic, Resistance | Different HIIT doses |
| Martinez et al. 2016 | Spain | RCT | 94 | mixed | 8.2 | NI | 12 | 2 | 10 to 20 | 120 | 20 | Circuit exercises | MICT and games |
| McManus et al. 1997 | United Kingdom | RCT | 30 | girls | 9.6 | NI | 8 | 3 | 10 and 30 | 30 and 90 | 20 | Running, sprint | Maintain daily life |
| McManus et al. 2005 | Hong Kong | RCT | 35 | boys | 10.3 | NI | 8 | 3 | 30 | 165 | 20 | Cycling, sprint | Maintain daily life |
| Moreau et al. 2017 | New Zealand | RCT | 305 | mixed | 9.9 | NI | 6 | 5 | 20 | 20 to 60 | 10 | Video workouts | Active control |
| Racil et al. 2013 | Tunisia | RCT | 34 | girls | 15.9 | Overweight  /Obese | 12 | 3 | 30 | 30 | 18 | Running | no exercise |
| Racil et al. 2016a | Tunisia | RCT | 75 | girls | 16.6 | Overweight  /Obese | 12 | 3 | 30 | 30 | 18 | Running | no exercise |
| Racil et al. 2016b | Tunisia | RCT | 47 | girls | 16.6 | Overweight  /Obese | 12 | 3 | 15 | 15 | 12 | Running | no exercise |
| Stenman et al. 2017 | Finland | RCT | 25 | mixed | 17.8 | NI | 2 | 4 | 60 | 75 | 19 | Circuit exercises | Maintain daily life |
| Tian et al. 2021 | China | RCT | 174 | mixed | 11 to 15 | NI | 24 | 3 | 60 | 30 | 10 | Mountain climbing | Light stretch |
| Williams et al. 2000 | United Kingdom | RCT | 45 | boys | 10 | NI | 8 | 3 | 10 and 30 | 30 and 90 | 14 | Cycling, sprint | Maintain daily life |
| Ricci et al. 2022 | United states | Cluster-RCT | 67 | mixed | 10.5 | NI | 6 | 1.5 | 20 | 30 | 19 | Circuit exercises | Regular PE |
| Williams et al. 2022 | United Kingdom | RCT | 16 | girls | 11.7 | NI | 2 | 3 | 10 | 50 | 6 to 8 | Running, sprint | Maintain daily life |
| Cao 2022a | China | RCT | 40 | mixed | 11 | Overweight  /Obese | 12 | 3 | 15 | 15 | 18 | Running | Maintain daily life |
| Cao 2022b | China | RCT | 45 | boys | 11.2 | Overweight  /Obese | 12 | 3 | 15 | 15 | 11 | Running | Maintain daily life |
| Harris 2022 | New Zealand | Cluster-RCT | 368 | mixed | 11 to 13 | NI | 16 | 2 | varied | varied | 15 | NI | Regular PE |
| Oliveira 2022 | United Kingdom | RCT | 19 | mixed | 13.2 | NI | 4 | 3 | 60 | 75 | 20 | Running | Maintain daily life |
| Bossmann 2022 | Germany | Cluster-RCT | 136 | mixed | 12.2 | NI | 6 | 2 | 60 or 240 | 40 or 160 | 20 or 28 | Running or circuit | Different HIITs |
| Overall | 17 | NA | 25104 | NA | 12.2 | 11 | 12 | 2.8 | NA | NA | NA | NA | NA |

N, number of participants; RCT, randomised control trial; NI, no information; PE, physical education; MICT, moderate-intensity continuous training; NA, not applicable.
