# Supplementary material for: Process evaluation of school-based high-intensity interval training interventions for children and adolescents: a systematic review and meta-analysis of randomized controlled trials
Source: BMC Public Health. 2024 Feb 2;24:348. doi: 10.1186/s12889-024-17786-6 (PMC10835840; doi:10.1186/s12889-024-17786-6)
Supplement: Supplementary file 5 — Additional file 5: Fig S1. Risk of bias assessment for cardiorespiratory fitness. Fig S2. Risk of bias assessment for body composition. Fig S3. Risk of bias assessment for muscular fitness. [file 12889_2024_17786_MOESM5_ESM.docx]

**Fig S1. Risk of bias assessment for cardiorespiratory fitness.** D1a, Randomisation process; D1b, Timing of identification or recruitment of participants; D2, Deviations from the intended interventions; D3, Missing outcome data; D4, Measurement of the outcome; D5, Selection of the reported result; low risk; some concerns; high risk; RCT, randomised controlled trial; YYIRT1, [Yo-Yo intermittent recovery test level 1; PACER, progressive aerobic cardiovascular endurance run.](https://www.scienceforsport.com/yo-yo-intermittent-recovery-test-level-1/)

**Fig S2. Risk of bias assessment for body composition.** D1a, Randomisation process; D1b, Timing of identification or recruitment of participants; D2, Deviations from the intended interventions; D3, Missing outcome data; D4, Measurement of the outcome; D5, Selection of the reported result; low risk; some concerns; high risk; RCT, randomised controlled trial; BMI (-z), body mass index (z score).

**Fig S3. Risk of bias assessment for muscular fitness.** D1a, Randomisation process; D1b, Timing of identification or recruitment of participants; D2, Deviations from the intended interventions; D3, Missing outcome data; D4, Measurement of the outcome; D5, Selection of the reported result; low risk; some concerns; high risk; RCT, randomised controlled trial.
