# Supplementary material for: Process evaluation of school-based high-intensity interval training interventions for children and adolescents: a systematic review and meta-analysis of randomized controlled trials
Source: BMC Public Health. 2024 Feb 2;24:348. doi: 10.1186/s12889-024-17786-6 (PMC10835840; doi:10.1186/s12889-024-17786-6)
Supplement: Supplementary file 7 — Additional file 7: Fig S4. Sensitive analysis by removing high risk of bias studies for CRF. N, participant number; SD, standard deviation; SMD, standard mean difference; CI, confidence interval; AEP, aerobic exercise programme; RAP, resistance and aerobic programme. Fig S5. Sensitive analysis by removing studies with computed outcome scores for cardiorespiratory fitness. N, participant number; SD, standard deviation; SMD, standard mean difference; CI, confidence interval. Fig S6. Funnel plot for studies reported cardiorespiratory fitness. Fig S7. Sensitive analysis by removing high risk of bias studies for body composition. N, participant number; SD, standard deviation; SMD, standard mean difference; CI, confidence interval; AEP, aerobic exercise programme; RAP, resistance and aerobic programme. Fig S8. Sensitive analysis by removing studies with computed outcome scores for body composition. N, participant number; SD, standard deviation; SMD, standard mean difference; CI, confidence interval. Fig S9. Funnel plot for studies reported body composition. Fig S10. Sensitive analysis by removing high risk of bias studies for muscular strength. N, participant number; SD, standard deviation; SMD, standard mean difference; CI, confidence interval; AEP, aerobic exercise programme; RAP, resistance and aerobic programme. Fig S11. Sensitive analysis by removing studies with computed outcome scores for muscular strength. N, participant number; SD, standard deviation; SMD, standard mean difference; CI, confidence interval. Fig S12. Funnel plot for studies reported muscular strength. [file 12889_2024_17786_MOESM7_ESM.docx]

Fig S4. Sensitive analysis by removing high risk of bias studies for CRF. N, participant number; SD, standard deviation; SMD, standard mean difference; CI, confidence interval; AEP, aerobic exercise programme; RAP, resistance and aerobic programme.

Removed studies (n = 14): Angel 2021, Cao 2022b, Cvetkovic 2018, Engel 2019, Fernandez 2019, Gamelin 2009, Ketlhut 2020, McManus 2005, Mucci 2013, Racil 2016a, Williams 2022, Baquet 2010, Larsen 2017, Ricci 2022.

Fig S5. Sensitive analysis by removing studies with computed outcome scores for cardiorespiratory fitness. N, participant number; SD, standard deviation; SMD, standard mean difference; CI, confidence interval.

Removed studies (n = 3): Costigan 2015, Lubans 2020, Takehara 2021.

Fig S6. Funnel plot for studies reported cardiorespiratory fitness.

Fig S7. Sensitive analysis by removing high risk of bias studies for body composition. N, participant number; SD, standard deviation; SMD, standard mean difference; CI, confidence interval; AEP, aerobic exercise programme; RAP, resistance and aerobic programme.

Removed studies (n = 11): Racil 2016a, Popowczak 2022, Nourry 2005, Engel 2019, Cvetkovic 2018, Cao 2022b, Boddy 2010, Abassi 2021, Baquet 2010, Baquet 2004, Win 2019.

Fig S8. Sensitive analysis by removing studies with computed outcome scores for body composition. N, participant number; SD, standard deviation; SMD, standard mean difference; CI, confidence interval.

Removed studies (n = 4): Lubans 2020, Costigan 2015, Takehara 2021, Popowczak 2022.

Fig S9. Funnel plot for studies reported body composition.

Fig S10. Sensitive analysis by removing high risk of bias studies for muscular strength. N, participant number; SD, standard deviation; SMD, standard mean difference; CI, confidence interval; AEP, aerobic exercise programme; RAP, resistance and aerobic programme.

Removed studies (n = 4): Baquet 2004, Cvetkovic 2018, Racil 2016a, Ricci 2022.

Fig S11. Sensitive analysis by removing studies with computed outcome scores for muscular strength. N, participant number; SD, standard deviation; SMD, standard mean difference; CI, confidence interval.

Removed studies (n = 2): Lubans 2020, Costigan 2015.

Fig S12. Funnel plot for studies reported muscular strength.
